# Effects of active travel interventions in the UK on disadvantaged population groups and their potential to reduce health inequalities: systematic review

**DOI:** 10.1136/jech-2026-225997

**Published:** 2026-04-28

**Authors:** Gareth J Hollands, Wendy Macdowall, Helen ED Burchett, Irene Kwan, Preethy D’Souza, Michelle Richardson, Claire Stansfield, David Ogilvie, Jenna Panter, James Thomas, Amanda J Sowden, Katy Sutcliffe

**Affiliations:** 1 EPPI Centre, UCL Social Research Institute, University College London, London, UK; 2 Faculty of Public Health and Policy, London School of Hygiene & Tropical Medicine, London, UK; 3 MRC Epidemiology Unit, University of Cambridge School of Clinical Medicine, Cambridge, UK; 4 Centre for Reviews and Dissemination, University of York, York, UK

**Keywords:** Health inequalities, PUBLIC HEALTH, PREVENTIVE MEDICINE

## Abstract

**Background:**

Differential impacts of active travel interventions on disadvantaged population groups have been under-studied. A review of evidence from the UK was commissioned to inform the development of government policy and support the implementation of equitable and effective interventions.

**Methods:**

Systematic review, identifying studies via searching 31 electronic databases. Eligible studies evaluated active travel interventions in the UK and reported quantitative data on active travel behaviours and/or linked health outcomes for population subgroups defined by sex/gender, ethnicity, age or socioeconomic status. Two reviewers screened reports, extracted data and assessed risk of bias. Outcome data were summarised in relation to directions of intervention effects for population subgroups.

**Results:**

We included 18 study reports collectively evaluating five interventions. Evidence suggests that interventions are more likely to increase than decrease active travel among females and may also be more likely to benefit females relative to males. Interventions are also more likely to increase than decrease active travel among older adults, but there is no clear evidence that they will benefit this group relative to younger adults. Available evidence gives no clear indication of likely positive or negative impacts on groups defined by ethnicity or socioeconomic status.

**Conclusion:**

There is some evidence that active travel interventions improve outcomes and may reduce inequalities in relation to sex, and improve outcomes among older adults. The available evidence for differential impacts of active travel interventions is otherwise largely inconclusive, but importantly provides no clear indication that implementing active travel interventions will widen inequalities.

**WHAT IS ALREADY KNOWN ON THIS TOPIC**

**WHAT THIS STUDY ADDS**

- The design of this review enabled assessments to be made concerning the extent to which active travel interventions in the UK are effective *within* typically less advantaged population subgroups—specifically females, older adults, minority ethnic groups and socioeconomically disadvantaged groups—as well as their effectiveness in these less advantaged subgroups *relative* to more advantaged subgroups. Its findings suggest that active travel interventions improve outcomes and may reduce inequalities in relation to sex and improve outcomes among older adults.

**HOW THIS STUDY MIGHT AFFECT RESEARCH, PRACTICE OR POLICY**

- This review provides no clear indication that implementing active travel interventions will widen inequalities and suggests that new research should be prioritised with funding that matches the costs of carrying out methodologically robust evaluations of these interventions.

## Introduction

 Active travel can be defined as walking, cycling, wheeling (with the use of mobility aids) or scootering activity, for the functional purpose of transport to a particular destination, such as work, school or the shops.^1 2^ Physical inactivity is a known risk factor for non-communicable disease.^3 4^ Accordingly, active travel and related physical activity have multiple health benefits including reduced risk of all-cause mortality, cardiovascular disease, diabetes, cancers, mental health conditions and dementia.^5–8^ Beyond benefits for population health, active travel also confers important cobenefits for planetary health.^9^ Substantial reductions in land transport emissions are needed to meet Net Zero commitments,^10^ and improvements to air quality produce health as well as environmental benefits. Furthermore, health inequities—unfair, socially produced and systematic disparities in health outcomes between population groups^11 12^—are entwined with active travel. Where socially and materially disadvantaged groups have lower or reduced prevalence of active travel, this has the potential to exacerbate inequalities in health, given adverse effects of physical inactivity. Lower population prevalence of active travel may also worsen inequalities via various indirect pathways.^13^ For example, people with lower socioeconomic status (SES) are more affected by the externalities of predominant non-active modes of travel. This includes being subject to disproportionate exposure to (and with a lesser role in producing) pollution and reduced safety and social connectivity resulting from motor vehicle use and its related infrastructure.^9 14–16^

The vital importance for population and planetary health of realising modal shifts towards active travel is not reflected in generally (although not uniformly) low levels of active travel seen in the UK. For example, in Great Britain in 2023, 330.8 billion vehicle miles were driven on roads, of which cycle traffic only made up 3.6 billion.^17^ Importantly, there is also a substantial body of evidence that levels of active travel differ by population groups, including those subject to disadvantage. In England in 2022, distance walked and cycled was negatively associated with increasing income quintile, and a lower distance (and involving fewer trips) was walked and cycled by those who had never worked and the long-term unemployed, versus those in managerial and professional occupations.^18^

Globally, a wide range of interventions have been implemented at varying scales to attempt to increase the uptake and prevalence of active travel behaviours, applying both individual and population-level approaches.^19 20^ These interventions have been widely evaluated via primary outcome evaluation studies and at the level of evidence syntheses, but this has principally been in relation to overall effects across populations. Where effects on specific population subgroups have been examined, this has predominantly concerned children and adolescents in relation to active school travel.^21^ Differential impacts of interventions for other population subgroups, particularly those subject to inequity and disadvantage, have been relatively understudied, despite such differences having the potential to reduce (or exacerbate) disparities in both levels of active travel and in health outcomes more generally. At present, there is weak evidence for positive health equity impacts of active travel interventions, and the potential for negative health equity impacts cannot be excluded.^22 23^ However, the value of previous reviews to support decision-making has been limited due to a combination of their relatively narrow scope in the population subgroups and related data they consider, their methodological quality and/or their age. Furthermore, the Department of Health and Social Care (England), who commissioned this review and were key stakeholders in its development, identified that previous reviews lacked a geographic focus relevant to England and the UK, meaning that they could not optimally inform national decision-making. Reflecting these factors, the current review—purposefully narrow in geographic scope but broad in its methodological scope—was commissioned to inform the ongoing development and implementation of active travel interventions and policies that are both effective and equitable. The wider policy context in England indicates that boosting active travel has continued to be a government priority across successive administrations, with notable recent actions including the formation of Active Travel England in 2020, publication of multiple Cycling and Walking Investment Strategies^24^ and explicit reference to active travel in key national health policy documents.^25^

The aim of this review was to assess the extent to which active travel interventions in the UK are effective *within* typically less advantaged population subgroups—specifically females, older adults, minority ethnic groups and socioeconomically disadvantaged groups—as well as their effectiveness in these less advantaged subgroups *relative to* more advantaged subgroups. These assessments can be used to inform understanding of the potential (in)equity impacts of active travel interventions, namely whether they are likely to improve or worsen outcomes among less advantaged people, and whether they are likely to reduce or increase inequalities between less advantaged and more advantaged people.

## Methods

We conducted a systematic review, reported in accordance with Preferred Reporting Items for Systematic Reviews and Meta-Analyses (PRISMA) and PRISMA-Equity^26^ and pre-registered on PROSPERO (CRD420251001436) and the Open Science Framework (OSF) (https://osf.io/hsdpg).

### Inclusion criteria

Eligible study reports evaluated active travel interventions and included quantitative data on active travel behaviours (eg, walking, cycling, composite active travel) and/or linked health outcomes, for population subgroup(s) defined by sex/gender, ethnicity, age or SES. Full eligibility criteria are detailed in table 1. We additionally included linked study reports that provided complementary contextual information on included interventions but that did not necessarily meet all the eligibility criteria, provided that intervention was represented by at least one eligible study report.

**Table 1:** Eligibility criteria

Population	**Population group(s) subject to disadvantage**Eligible populations comprised specific disadvantaged groups, or general populations where outcomes were reported separately for at least one population subgroup subject to disadvantage (see also ‘outcome’) relating to the following dimensions of equity: sex/gender, age, ethnicity and socioeconomic status (with categorisation via, eg, education, income, geography). Populations needed to be located in England and/or the UK. We excluded children and adolescents in relation to active school travel as this context was of lesser priority to commissioners given the predominant focus it had received in the prior literature.
Intervention	**Any intervention that focuses on increasing active travel**Eligible interventions were those with a focus on increasing active travel. Active travel is defined as walking, cycling, wheeling (the use of mobility aids) or scootering activity, with the purpose of transport to a particular destination (ie, getting from place to place), such as to work or the shops. Studies must have indicated that the intervention focused on, or aimed to increase, active travel, either explicitly described in its intervention aims or content, or strongly suggested by its context (eg, altering commuting, transport or travel behaviour) and/or its inclusion of active travel outcomes. Eligible interventions may also include aims or components related to travel or physical activity for non-transport purposes (eg, recreation or leisure, sport, fitness, exercise, performance or rehabilitation) providing they do not constitute the predominant focus of the intervention, and/or are not implemented in contexts explicitly related to these kinds of activity (eg, organised or prescribed sport or exercise programmes in leisure facilities). We excluded interventions that focused mostly on altering uptake or related aspects of other forms of transport (eg, public transport provision or usage incentive schemes, or speed limits or economic disincentives for motor vehicles) without at least equivalent actions for enabling active travel as defined (eg, cycle training, physical infrastructure).
Comparison	**Absence of the active travel intervention**Eligible comparisons were the absence of the intervention (including lower relative levels of exposure in terms of proximity to its implementation), which could include comparison to groups not exposed to the intervention or to the same population at different timepoints preimplementation and postimplementation in longitudinal designs.
Outcome	**Quantitative measure of active travel behaviour or linked health outcome**Eligible outcomes were quantitative measures of active travel behaviour or health outcomes linked to performance of these behaviours, reported for at least one eligible population subgroup. This could be (1) a specified group subject to disadvantage or (2) a subgroup(s) derived from a general population. Among general population studies, outcome data could be reported by specific subgroup(s), or represented by an interaction term in a statistical model with a statement specifying the direction of effect on that group(s). Active travel could be assessed via self-report, observation or an objective measure of participants’ behaviour (eg, accelerometer, geographic sensor (eg, Global Positioning System)). Health outcomes could be assessed via self-report or objective measures of health or disease status, or physiological or psychological functioning, including quality of life and mental health. Should outcome data linked specifically to active travel not be available, we considered including physical activity data providing that first, we could reasonably infer it likely that active travel makes up a major proportion of the physical activity measure (informed by, eg, the content of measures used, the intervention context and available complementary information), and second, where the intervention’s implementation and assessment context was such that the purpose of any observed physical activity (eg, for transport) was likely not feasible to directly assess.
Study design	**Evaluation using a randomised, longitudinal or quasi-experimental design**Any evaluative design was eligible, eg, randomised, longitudinal (eg, simple pre/post comparison, interrupted time series) or other quasi-experimental design (eg, non-randomised controlled trial, controlled before-and-after) when applied to data collection for a specified intervention or policy (or set of interventions/policies).
Publication type	**Primary research reports**Empirical reports of primary research studies. ie, not a protocol, study registration or otherwise not a full journal or report article.

### Search strategy

Study reports were identified from electronic searches of 31 databases from inception, current to 7 March 2024, supplemented by searching reference lists of included reports and relevant systematic reviews and contacting authors of included studies. Details of searches are provided in online supplemental material S1.

### Selecting eligible studies

Bibliographic records were imported into EPPI Reviewer^27^ and duplicates discarded. In a pilot screening phase, a high degree of average agreement (97.4%) on inclusion/exclusion decisions for a random sample of 1000 records was achieved between reviewers. Title–abstract screening was then conducted by a single reviewer, with a second reviewer involved if necessary to reach a decision. An active learning algorithm (‘Priority screening’ tool in EPPI Reviewer) was used to prioritise title–abstract records for manual screening, whereby unscreened records are continually reprioritised by a machine learning classifier trained to distinguish between eligible and ineligible records based on the growing corpus of decisions. Title–abstract screening was truncated in consultation with the information specialist at a point where inclusion rates had plateaued such that the resource cost of continuing screening could not be justified. Full-text reports of all potentially eligible records were screened independently by two reviewers, with any inability to reach consensus resolved by consulting a third reviewer as arbiter.

### Data extraction and synthesis

Two reviewers extracted and checked information on key study characteristics and outcome data, and used the Effective Public Health Practice Project (EPHPP) quality assessment tool^28 29^ to independently assess risk of bias (termed quality assessment in the EPHPP tool) before reaching agreement by consensus. The EPHPP tool has been extensively employed in other active travel reviews.^2 20 30 31^ It assesses six dimensions: selection bias, study design, confounders, blinding, data collection methods and withdrawals and dropouts, with each dimension rated as either strong, moderate or weak. An overall rating for each evaluation is calculated on the same 3-point scale, with a score of weak overall if at least two dimensions were rated as weak, moderate overall if one dimension was rated as weak and strong overall if no dimension was rated as weak. In line with other active travel reviews, we recognised the need to adapt the tool for active travel intervention evaluations.^20 30 31^ We provided additional clarification on how to assess some criteria (eg, selection bias Q1) and amended the answer categories for some questions (eg, selection bias Q2), to better reflect that for evaluations of large infrastructure interventions, it is unlikely to be feasible to achieve a high response rate from a general population sample. Furthermore, where additional papers reported evaluation methods (eg, protocols), these were also used to complete the assessment.

Our treatment of equity in the synthesis process was informed by ongoing development of a checklist being developed within the London-York PRP Evidence Review Facility to help guide consideration of issues of health equity in evidence synthesis.^32^ Completing this checklist guided our approach to identifying, synthesising and reporting evidence in relation to specified population subgroup foci (see online supplemental material S5 for further details). We had intended to also use the complementary PRO-EDI tool,^33^ but at the time of completing this review, it was not yet available. We also report the review in accordance with PRISMA-Equity.^26^

Our approach to synthesis was to, first, extract any evidence of intervention effectiveness for the specified population subgroups, namely sex/gender, age, ethnicity and SES (with subcategorisation of SES by area-based deprivation, educational level, housing status/tenure, household income, employment status and social grade). We extracted evidence for each of three behavioural outcomes: walking, cycling and composite active travel, as well as for linked health outcomes. When necessary, we contacted study authors for additional data. We were unable to conduct any meta-analyses due to heterogeneity of the usable data. We therefore sought to extract all eligible outcome data that allowed assessment of intervention effectiveness in typically less advantaged population subgroups. This meant that we extracted data where available for multiple measures of walking, cycling or composite active travel (as opposed to selecting a single measure), and that these data related to different ways of defining and categorising population subgroups. As per the protocol, we extracted data from the longest available timepoint for any given outcome. The relevant numeric data and our interpretation of those data for any given result were systematically tabulated. Because our focus was on intervention effects on population subgroups, we did not systematically extract or attempt to synthesise quantitative data on the main effects of interventions.

Outcome data were then visually represented in an effect direction plot and summarised narratively, principally in relation to directions of effects rather than their statistical significance (as per guidance for synthesising evidence without meta-analysis).^34^ We did not simply count directions and instead systematically assessed whether for each specified population subgroup, there were any consistent directions of intervention effects on: (1) each outcome across all intervention evaluations and (2) for those outcomes within each specific intervention evaluation. In accordance with the aims of the review, this approach was applied to both evidence of effects within less advantaged population subgroups specifically, as well as effects in less advantaged relative to more advantaged subgroups.

## Results

### Results of the search

**Figure 1:**
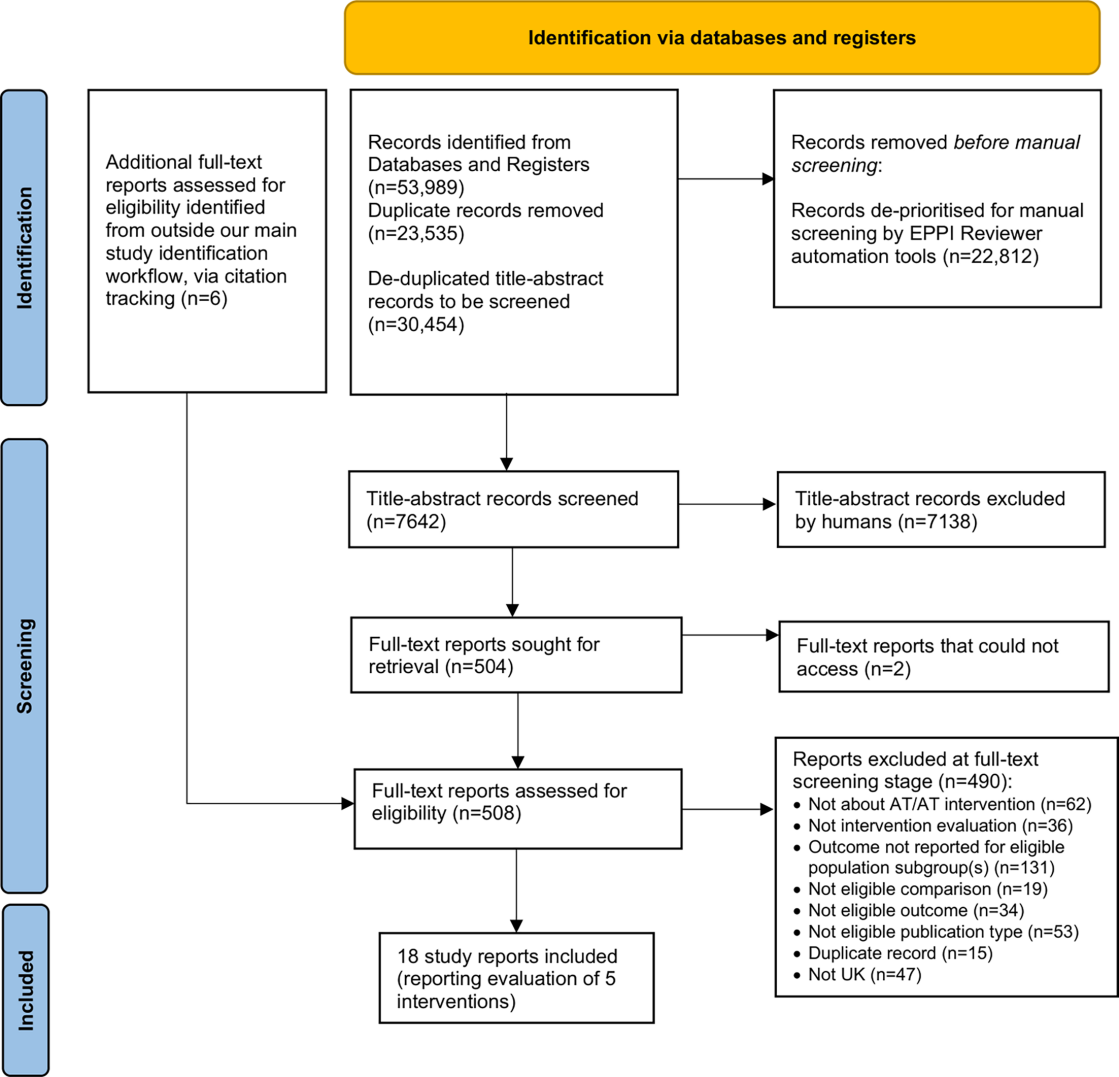
Preferred Reporting Items for Systematic Reviews and Meta-Analyses (PRISMA) flow diagram.

Details of the identification process are shown in the PRISMA flow diagram (figure 1). Screening of 7642 title–abstract records, followed by assessment of 508 full-text reports, resulted in 18 study reports that met the inclusion criteria. These collectively evaluated five active travel interventions.

### Characteristics of included study reports

Key characteristics of the included interventions and their evaluations are reported in table 2. All five interventions—denoted respectively with the names ‘Cambridgeshire Guided Busway’; ‘Cycling Towns’; ‘ENABLE London’; ‘iConnect’; and ‘Mini-Hollands’—consisted of major programmes of infrastructure development in the UK (mainly England), which aimed to increase active travel. Cycling Towns was the only intervention that also included a range of other activities aimed at promoting behaviour change. More comprehensive details of the interventions following TIDieR guidance^35^ and a summary of their behavioural components (behaviour change techniques)^36^ are provided in online supplemental material S2.

**Table 2:** Characteristics of included interventions and evaluations

Name (evaluation reports*)	Population	Intervention†	Comparison	Outcomes	Design; quality assessment
**Cambridgeshire Guided Busway** (*primary reports:* Panter *et al* 2016,^42^ plus data from author correspondence; *additional linked reports*: Heinen *et al* 2015^45^; Heinen *et al* 2015^46^; Ogilvie *et al* 2010^44^; Panter *et al* 2011^43^	People aged ≥16 years who worked in areas of Cambridge to be served by the busway and lived within ~30 km of the city *Available data by subgroups:* Sex/gender Age SES (educational level, housing status)	A new bus network and an adjacent 22 km traffic-free walking and cycling route	Individual level change in outcomes between baseline and follow-up, using a measure of proximity to the nearest busway stop or path access point to represent exposure to the intervention	**Active travel**: weekly time spent in active commuting (in minutes) categorised as ‘no change’; ‘increase’; ‘decrease’ *Data collection/source:* postal surveys; self-reported data using validated past 7-day recall instrument	Cohort: single group pre + post; Weak
**Cycling towns** (*primary reports*: Patterson *et al* 2023^52^ for evaluation 1; Sloman *et al* 2009^53^ for evaluation 2; *Additional linked reports*: Goodman *et al* 2013^54^	People aged ≥16 years, employed and living in the same LA area at both time points in 18 intervention towns *Available data by subgroups:* Sex/gender Age SES (area-based deprivation, educational level)	Cycle lanes and tracks in 6 CDTs and 12 cycling cities and towns, also included a range of behavioural components	Individual level change in outcomes between 2001 and 2011, using a matched comparison group‡ (largest urban region within the English LA most similar to each intervention LA)	**Walking**: as usual commute mode **Cycling**: as usual commute mode; taking up cycling; maintaining cycling **Active travel**: cycling or walking as usual commute mode *Data collection/source:* longitudinally linked census data in the ONS Longitudinal Study of England and Wales§	Cohort analytic: two group pre + post; Strong
	People aged ≥16 years, living in 6 CDTs *Available data by subgroups:* SES (social grade)		Change in proportion reporting outcomes between baseline and follow-up	**Cycling:** any cycling in a typical week in the previous year *Data collection/source:* telephone *s*urveys, self-reported data	Other: single group with different cohort pre + post; Moderate
**ENABLE London** (*primary reports:* Limb *et al* 2020^37^; Owen *et al* 2020^38^‡ *Additional linked reports*: Nightingale 2019 *et al*^39^; Ram *et al* 2016^40^	People aged ≥16 years residing in Greater London (largely from East London and the London Borough of Newham) who moved into social, intermediate and market-rent accommodation in East Village (part of the regeneration of the former 2012 London Olympic site) *Available data by subgroups:* SES (housing status)	Purpose-built, mixed-use residential development specifically designed to encourage healthy active living by improving walkability and access to public transport	Individual-level change in outcomes between baseline and follow-up, using a comparison group of people who were seeking to move to East Village at baseline but had not at the time of follow-up	**Walking:** daily walking (in minutes) **Cycling:** daily cycling (in minutes) **Active travel:** daily walking/cycling (in minutes) **Health:** BMI and percentage fat mass *Data collection/source:* self-complete computer-assisted personal interviews in participants’ homes, researcher taken measures of height, weight and bioimpedance plus accelerometer and a GPS monitor to investigate movements for 7 consecutive days	Cohort analytic: two group pre+post; Moderate
**iConnect** (*primary reports:* Goodman *et al* 2014^48^; Panter *et al* 2017^47^; Song *et al* 2017,^49^ plus data from author correspondence; *Additional linked reports*: Ogilvie *et al* 2012^50^; Sahlqvist *et al* 2013^51^	People agedv≥18 years living within 5 km by road of the Connect2 projects *Available data by subgroups:* Sex/gender Age Ethnicity SES (educational level, household income, employment status)	Infrastructure projects aimed at making walking and cycling easier at ~80 sites around the UK, of which three (in Cardiff, Kenilworth and Southampton) were evaluated	Individual-level change in outcomes between baseline and follow-up, using a measure of proximity to the nearest access point to the Connect2 infrastructure to represent ‘more’ or ‘less’ exposure to the intervention	**Walking**: walking for transport; short-lived increase in walking for transport; sustained increase in walking for transport; uptake of walking for transport **Cycling**: cycling for transport **Active travel:** modal shift from private motor vehicles to walking/cycling in terms of time share and distance share *Data collection/source:* postal surveys; self-reported data using past 7-day recall instrument	Cohort: single group pre + post; Weak
**Mini-Hollands** (*primary reports:* Aldred *et al* 2019,^41^ plus data from author correspondence)	People aged ≥16 years, living in three ‘mini-Holland’ Outer London boroughs *Available data by subgroups:* Sex/gender Age Ethnicity SES (employment status)	Redesigned town centres, with cycle hubs at stations; motor traffic calming and reduction measures in residential areas; and physically protected cycle lanes along main roads	Individual-level change in outcomes among those living in ‘high dose’ mini-Holland areas between baseline and follow-up, using a comparison group of people from ‘low dose’ and non mini-Holland Outer London boroughs	**Cycling:** past week cycling (in minutes) **Active travel**: past week active travel (in minutes) *Data collection/source:* online survey; self-reported data using past 7-day recall instrument	Cohort analytic: two group pre + post; Weak

* Reports of intervention evaluations that contained data that we used for our main analyses are termed ‘primary reports’. ‘Additional linked reports’ are related reports that informed other aspects of the review process. eg, compiling the TIDieR table, providing complementary contextual information on included interventions and/or reporting less comprehensive data that overlapped with that available in ‘primary reports’.

† See TIDieR table (online supplemental material S2) for a full description of the interventions.

‡ We used this comparison, but the evaluation also included two other comparison groups: an unfunded comparison group and a non-London national comparison group.

§ The ONS Longitudinal Study contains linked census and life events data for a 1% sample of the population of England and Wales.

BMI, body mass index; CDTs, cycling demonstration towns; GPS, Global Positioning System; LA, local authority; SES, socioeconomic status.

The ENABLE London^37–40^ and Mini-Hollands^41^ evaluations used a two-group cohort study design (ie, an intervention and comparison group, with the same people within each of those groups providing data at baseline and follow-up). Cambridgeshire Guided Busway^42–46^ and iConnect^47–51^ evaluations used a single-group cohort study design (ie, the same group of people within a single group providing data at baseline and follow-up); although in these there was no external comparison group, quasi-experimental analysis methods were employed in which proximity to the infrastructure developments represented exposure to the intervention. Cycling Towns^52–54^ was evaluated using two different methodological approaches: a two-group cohort study design^52^ and a single group non-cohort design (ie, different samples of people provided data at baseline and follow-up).^53^

The evaluations of all five interventions involved self-reported data collected via surveys. The mode of survey delivery varied and included: face-to-face computer-assisted personal interviews (ENABLE London); postal surveys (Cambridgeshire Guided Busway and iConnect); and online surveys (Mini-Hollands). Cycling Towns used telephone surveys^53^ and drew on existing survey data, namely longitudinally linked census data in the ONS Longitudinal Study of England and Wales^52^ in its two evaluations, respectively. ENABLE London also included accelerometer and Global Positioning System monitoring to investigate participants’ movements. All evaluations included measures of active travel including walking (for transport); cycling (for transport); or a composite of walking and/or cycling (for transport); however, the nature of these measures varied considerably. Only the ENABLE London evaluation included measures of health outcomes (body mass index (BMI) and % body fat). Participants in all evaluations were aged 16 years and over, apart from those in iConnect, who were aged 18 years and over.

### Risk of bias assessment

We conducted six risk of bias (framed as ‘quality’ by the EPHPP tool) assessments, since one intervention (Cycling Towns) had two distinct evaluations that used different methods and data sources.^52 53^ One evaluation was rated as strong overall (Cycling Towns)^52^, one as moderate (ENABLE London) and four as weak (Cambridgeshire Guided Busway; Cycling Towns^53^; iConnect; Mini-Hollands). For further details on risk of bias assessment, see online supplemental material S3.

### Assessment of equity-related impacts

**Figure 2:**
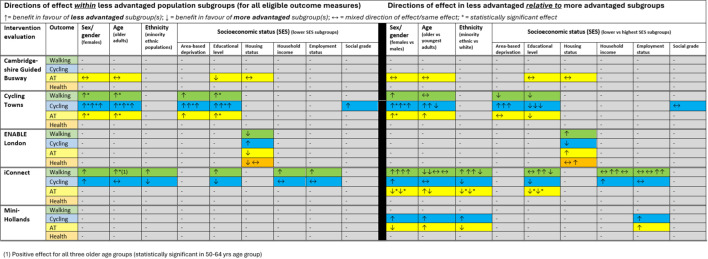
Directions of intervention effects by population subgroups. Reflecting the research questions, this summarises both (1) intervention effects within typically less advantaged population subgroups (left hand plot) and (2) intervention effects in typically less advantaged relative to more advantaged subgroups (right hand plot). AT, active travel; SES, socioeconomic status.

Directions of intervention effects by intervention evaluation and equity dimension for each outcome are presented in figure 2, with the underlying data tabulated in online supplemental material S4.

In table 3, we narratively summarise these effects, for each specified equity dimension (ie, sex/gender, age, ethnicity, SES).

**Table 3:** Summary of equity-related impacts by dimensions of equity

Equity dimension	What evidence is available?	What are the effects of these interventions *within* less advantaged population subgroups?	What are the effects of these interventions in less advantaged subgroups *relative to* more advantaged subgroups?	Summary
**Sex/gender**	Four intervention evaluations (Cambridgeshire Guided Busway; Cycling Towns; iConnect; Mini-Hollands) examined active travel outcomes according to sex/gender (all using binary categorisations of sex, ie, females/males). Of these, three (Cambridgeshire Guided Busway; Cycling Towns; iConnect) included an examination of intervention effects specifically among females, and all four (Cambridgeshire Guided Busway; Cycling Towns; iConnect; Mini-Hollands) examined intervention effects among females *relative to* males.	*What are the effects of these interventions among females?* For intervention effects specifically within female population subgroups, there was overall most evidence for positive effects and no evidence of negative effects. Two intervention evaluations (Cycling Towns; iConnect) showed consistently positive effects among females for walking, cycling and composite active travel, some of which were statistically significant, while the third (Cambridgeshire Guided Busway) showed no consistent direction of effect for composite active travel.	*How do outcomes compare between females and males?* For intervention effects in female relative to male subgroups, the evidence was mixed overall, but with more evidence of effects benefiting (as opposed to disbenefiting) females. Three evaluations (Cycling Towns; iConnect; Mini-Hollands) showed effects consistently benefiting females for walking and/or cycling, but effects for composite active travel were mixed, with one evaluation (Cambridgeshire Guided Busway) showing no consistent direction of effect, one (Cycling Towns) showing effects benefiting females and two (iConnect; Mini-Hollands) showing effects disbenefiting females.	The available evidence suggests that active travel interventions are more likely to improve than worsen active travel outcomes among female populations and may be more likely to reduce than increase inequalities between females and males. There is no clear evidence to suggest that active travel interventions will worsen outcomes for females or exacerbate inequalities in relation to sex/gender.
**Age**	Four intervention evaluations (Cambridgeshire Guided Busway; Cycling Towns; iConnect; Mini-Hollands) examined active travel outcomes according to age. Of these, three (Cambridgeshire Guided Busway; Cycling Towns; iConnect) included an examination of intervention effects specifically among older adults and all four (Cambridgeshire Guided Busway; Cycling Towns; iConnect; Mini-Hollands) examined intervention effects among older adults *relative to* the youngest adults.	*What are the effects of these interventions among older adults?* For intervention effects specifically within older adult population subgroups, there was overall most evidence for positive effects and no evidence of negative effects. One intervention evaluation (Cycling Towns) showed consistently positive effects among older adults for walking, cycling and composite active travel, some of which were statistically significant; a second (iConnect) showed positive effects for walking and no consistent direction of effect for cycling, while the third (Cambridgeshire Guided Busway) showed no consistent direction of effect for composite active travel.	*How do outcomes compare between older and younger adults?* For intervention effects in older adult relative to the youngest adult subgroups, there was overall no clear evidence for effects either benefiting or disbenefiting older adults. One intervention evaluation (Mini-Hollands) showed effects benefiting older adults for cycling and composite active travel. Effects for the other three (Cambridgeshire Guided Busway; Cycling Towns; iConnect) were mixed in that there were no consistent directions of effects either within each of those intervention evaluations or across walking, cycling and composite active travel outcomes.	The available evidence suggests that active travel interventions are more likely to improve than worsen active travel outcomes among older adult populations, although there is no clear evidence that they will either reduce or increase inequalities between older and younger adults. There is no clear evidence that active travel interventions will worsen outcomes for older adults or exacerbate inequalities in relation to age.
**Ethnicity**	Two intervention evaluations (iConnect; Mini-Hollands) examined active travel outcomes according to ethnicity. Both summarised and presented data in relation to white/non-white categorisations with the non-white category representing ‘Black, South Asian, mixed race and ‘other’ ethnic groups’ (iConnect) and ‘Black, Asian and Ethnic Minority groups’ (Mini-Hollands). Of these evaluations, one (iConnect) included an examination of intervention effects specifically among minority ethnic (non-white) populations, and both (iConnect; Mini-Hollands) examined intervention effects among non-white populations *relative to* white populations.	*What are the effects of these interventions among minority ethnic (non-white) populations?* For intervention effects specifically within non-white populations, the overall evidence was unclear. One intervention evaluation (iConnect) showed increases in walking among non-white populations, but decreases in cycling.	*How do outcomes compare between non-white and white populations?* For intervention effects in non-white populations relative to white populations, there was overall no clear evidence for effects either benefiting or disbenefiting non-white populations. Effect directions were mixed both within each intervention evaluation (iConnect; Mini-Hollands) as well as across walking, cycling and composite active travel outcomes.	The available evidence does not give a clear indication of whether active travel interventions are more likely to improve or worsen active travel outcomes among people from any minority ethnic populations, nor whether they will either reduce or increase inequalities between different ethnic groups. However, there is no clear evidence that active travel interventions will worsen outcomes or exacerbate ethnic inequalities.
**SES**	All five intervention evaluations examined active travel outcomes (as well as health outcomes of BMI and body fat in ENABLE London) according to at least one indicator of SES. Of these, four included an examination of intervention effects specifically among people with indicators of lower SES, including in relation to area-based deprivation (Cycling Towns); educational level (Cambridgeshire Guided Busway; Cycling Towns; iConnect); housing status/tenure (Cambridgeshire Guided Busway; ENABLE London); household income (iConnect); employment status (iConnect); and social grade (Cycling Towns). All five examined intervention effects among people with indicators of lower SES relative to those with indicators of higher SES. This was examined in relation to area-based deprivation (Cycling Towns); educational level (Cambridgeshire Guided Busway; Cycling Towns; iConnect); housing status/tenure (Cambridgeshire Guided Busway; ENABLE London); household income (iConnect); employment status (iConnect; Mini-Hollands); and social grade (Cycling Towns).	*What are the effects of these interventions among socioeconomically disadvantaged populations?* For intervention effects specifically within lower SES population subgroups, the overall evidence was unclear. One intervention evaluation (Cycling Towns) showed consistently positive effects for walking, cycling and composite active travel, some of which were statistically significant. Evidence from the other three interventions (Cambridgeshire Guided Busway; ENABLE London; iConnect) was mixed, with positive and negative or no consistent directions of effects both within each of those intervention evaluations as well as across walking, cycling, composite active travel and health outcomes.	*How do outcomes compare between socioeconomically disadvantaged and advantaged populations?* For intervention effects in people with lower SES relative to people with the highest SES, one intervention evaluation (Mini-Hollands) showed effects favouring people with lower SES for both cycling and composite active travel, but in the other four (Cambridgeshire Guided Busway; Cycling Towns; ENABLE London; iConnect) the evidence was mixed, with positive and negative and/or no consistent directions of effects, both within each intervention evaluation as well as across walking, cycling, composite active travel and health outcomes.	The available evidence does not give a clear indication whether active travel interventions are more likely to improve or worsen active travel and health outcomes among people with lower SES, nor whether they will either reduce or increase inequalities between people with lower SES and people with the highest SES. However, there is no clear evidence that active travel interventions will worsen outcomes or exacerbate inequalities in relation to SES.

BMI, body mass index; SES, socioeconomic status.

## Discussion

This review aimed to assess the extent to which active travel interventions in the UK are effective within less advantaged population subgroups specifically, as well as relative to more advantaged subgroups. The existing evidence suggests that such interventions are more likely to increase than decrease active travel among females and also among older adults, and may also be more likely to benefit females relative to males, but with no clear evidence that they will benefit older adults relative to younger adults. Regarding minority ethnic and socioeconomically disadvantaged groups, the available evidence is inconclusive.

The inconclusive nature of much of this evidence base stems from multiple factors. This review was commissioned by national-level policymakers to identify and synthesise evidence solely from the UK, with the benefit of being able to more directly inform the development of national strategies concerning active travel. However, this resulted in relatively few intervention evaluations being eligible for inclusion, which limited the extent of data to be synthesised and the ability to discern and interpret clear patterns of effects for any specific equity indicator or outcome. This issue was compounded by mixed and inconsistent effects and substantial heterogeneity in the characteristics of included evaluations and their reporting. Heterogeneity included the geographic contexts which varied at the micro-level and macro-level, active travel outcomes being assessed in many ways (eg, by uptake, frequency or distance, over differing time frames), and treatment of equity dimensions (eg, socioeconomic disadvantage being assessed by several different indicators or age being categorised using different age range brackets). The observation that this evidence base is highly heterogenous and thus restricts the ability to draw definitive inferences is one frequently noted in the wider active travel literature.^30 55 56^

Despite the complex and mostly inconclusive overall picture, the available evidence does suggest that active travel interventions likely improve outcomes and may reduce inequalities in relation to people’s sex. This is broadly consistent with non-UK studies finding that environmental infrastructure interventions increase active travel uptake in females, although with similarly limited and inconsistent evidence.^56^ It also suggests that such interventions may improve outcomes for older adults. The wider literature pertaining to impacts on older age groups is even more limited than that for sex/gender, but in accordance with our results, it has been found that a majority of active travel interventions benefit older adults.^57^ Importantly, the available evidence provides no clear indication that implementing such interventions will worsen outcomes among disadvantaged populations, or widen inequalities.

### Strengths and limitations

Strengths of this review include its wide methodological scope. It focused on relatively understudied dimensions of equity in relation to active travel interventions, drawing on a wide range of data and supported by comprehensive literature searches and robust study identification and data extraction processes (including multiple author contacts to obtain data). Furthermore, the review was commissioned by, and its protocol developed in consultation with, national-level policymakers to ensure it was designed to be optimally useful in informing current and developing UK government policy on active travel implementation and evaluation.

There are also several limitations. While its commissioners purposefully limited the review to UK evidence to increase its relevance for informing national policy and research, this likely limits its generalisability to, as well as its ability to derive useful insights from, other geographic regions. There are also limitations conferred as a result of the methods we used. We sought to extract all outcome data that allowed assessment of intervention effectiveness in typically less advantaged population subgroups—a necessarily pragmatic approach to maximise the likelihood that the review could usefully inform policymakers, rather than limiting inclusion and analysis to more selective outcomes and reducing heterogeneity but risking a literally or essentially empty review.^58^ This approach resulted in, for example, collating measures which had been operationalised in different ways, both within and between intervention evaluations, and sometimes multiple interdependent effects within evaluations, and then treating observed directions of effect as broadly comparable. This approach limits our ability to interpret overall effects (eg, assessing their strength) beyond high-level summaries of the consistency of effect directions across equity indicators, interventions and outcomes. Otherwise, any single effect risks being overinterpreted, as the apparent effect may be small or imprecise, or substantially different in nature from another that it appears comparable to. In addition, while we were relatively inclusive in terms of active travel outcomes, we did not include additional outcomes closely related to active travel that may have allowed us to draw on a wider range of evidence, in particular, the uptake of public transport (which typically involves some unavoidable active travel). A final key limitation is that we did not extract or synthesise data on the main effects of interventions at the level of the overall, general population. This was due to our focus being on interventions’ effects on specified population subgroups, guided by the priorities of the commissioners of the research. However, main effects of interventions can mask important equity impacts, and, in turn, equity-related effects do not necessarily indicate effects at the general population level. Accordingly, explicitly linking these respective data may have provided useful additional context for understanding the implications of our findings for the implementation and evaluation of active travel interventions for the general populations that they commonly target.

### Implications for policy, practice and research

This review has implications for guiding more robust and consistent assessment of potential equity impacts of active travel interventions. The current UK evidence is limited in quantity and is largely inconclusive, suggesting a need to prioritise new research. Typically, the responsibility for implementing major active travel (eg, infrastructural) interventions at scale rests with national and local government, and in the UK there have been recent increases in funding provision. For example, the UK Government’s 2025 Spending Review provided substantial funding for Active Travel England, an executive agency supporting local authorities in delivering and evaluating active travel interventions. Funding that matches the actual costs of carrying out methodologically robust evaluations of these interventions will produce higher quality outcome data. Large-scale evaluations typically involve collecting large quantities of original primary data because routinely collected active travel data are rarely of sufficient quality and detail for most evaluation purposes. Evaluations designed to investigate equity impacts are likely to generate additional costs, with quantitative analyses of population subgroups requiring larger samples to ensure representativeness and oversampling needed to account for dropout. Additional costs will also be associated with extended durations of follow-up needed to capture behavioural and health impacts that potentially accrue over time. To ensure evaluations are prioritised, initiatives to increase collaboration as well as the bidirectional transfer of expertise and resource between policy and research contexts should be encouraged, such as between local authorities and research institutions.^59–61^

Beyond the relative scarcity of relevant research in the UK, this review has identified some specific gaps in the evidence base and limitations that, if addressed, would increase the usefulness of future data. First, concerning equity, there was particularly limited data concerning impacts on ethnic minority populations, limiting our ability to explore differences between different ethnic groups. Second, all evaluated interventions were largely infrastructural, meaning that there is a lack of evidence about the effects of interventions delivered specifically to less advantaged individuals or groups. This includes evaluations able to estimate the contribution of specific behaviour change techniques or components, which also depends on evaluations being comprehensively designed and reported to allow effects of specific components to be disentangled.^35 36^ However, the current predominant focus on infrastructural interventions may be considered justifiable, given that personal safety concerns linked to a lack of safe infrastructure are consistently found to be the most important barriers to people engaging in active travel,^62^ with evidence that women and ethnic minorities may be especially discouraged.^63^ Furthermore, such environmental interventions requiring less agency from the recipient have greater potential to change behaviour at scale and are more likely to contribute to reducing inequalities,^64–67^ with accumulating evidence that they are typically more potent than more agentic, informational interventions.^68–70^ A final notable evidence gap is the lack of linked health outcome data, with only one evaluation reporting such data (ENABLE London, providing data on BMI and body fat). However, collecting health-related outcomes is unlikely to be practicable or indeed an appropriate priority for most active travel intervention evaluations.^71^

In terms of limitations to be addressed in future research with a focus on equity impacts, active travel evaluations should collect, report and explicitly interpret numeric data to specify impacts on population subgroups. The lack of usable data available for this review may in part be due to the focus on reporting main effects rather than any differential equity effects, possibly because of sample size requirements and/or such effects being small, and so at risk of being overinterpreted. However, equity data should still be made available to enable accumulation of evidence beyond individual study reports. This observation reflects wider patterns in public health evaluations concerning the lack of available data to allow investigation of equity.^12^ This could be addressed through expanding current initiatives to support and motivate researchers conducting primary studies to collect, report or make equity data available. This could be achieved, for example, by mandating that outcome data are disaggregated by equity-relevant variables.^72^ This would also facilitate increased curation and use of individual participant level data from primary research studies, thus increasing the potential for more powerful analyses.^73^ Second, more consistency in how equity dimensions are conceptualised and assessed would be beneficial. While challenging to address because standardised measurement is not necessarily appropriate across all characteristics and contexts, this may be aided by formal guidance to encourage systematic consideration and reporting of equity,^74 75^ as well as the aforementioned mandation of consistent equity data.

A continued obstacle to building a cumulative evidence base is inconsistency in assessing active travel outcomes, but addressing this is highly challenging. While improved guidance could lead to some improvements in consistency, such as more standardised wording for questionnaire measures, true standardisation (eg, core outcome sets) appears both unworkable and unlikely to be desirable. Active travel interventions as well as people’s behaviours are highly contextual and complex, and the heterogeneity of outcome measures reflects that. Specific evaluations of these interventions benefit from integrating and triangulating multiple different types of data to build a more contextual and nuanced combination of evidence of the intervention’s effects.^76^ To take an example from the current review, from the iConnect evaluation, we included data on walking that related to different trajectories of effects, data on cycling that related to aggregate effects and data that related to modal shifts towards active travel behaviours. These different types of data each address specific and important elements of the intervention’s effects, and so increasing consistency and homogeneity of assessment could limit the depth of insights from primary evaluations.

In conclusion, due to the sparsity of evaluation studies, inconsistent effects and heterogeneity in contexts, outcome measures and treatment of equity, the available evidence for the UK is largely inconclusive. There is, however, some evidence that active travel interventions improve outcomes and may reduce inequalities in relation to sex/gender, as well as improve outcomes among older adults. Importantly, the available evidence provides no clear indication that implementing such interventions will widen inequalities.

## Supplementary material

10.1136/jech-2026-225997 online supplemental file 1

## Data Availability

All data relevant to the study are included in the article or uploaded as supplementary information.
