# Supplementary material for: Effects of active travel interventions in the UK on disadvantaged population groups and their potential to reduce health inequalities: systematic review
Source: J Epidemiol Community Health. 2026 Apr 28;80(9):e225997. doi: 10.1136/jech-2026-225997 (PMC13479686; doi:10.1136/jech-2026-225997)
Supplement: Supplementary file 1 [file jech-80-9-s001.docx]

## **Supplementary materials**

**Contents**

S1: Search strategy

S2: Description of evaluated interventions

S3: Risk of bias assessment

S4: Data underlying effect direction plot (Figure 2 in main paper)

S5: Use of London-York PRP Evidence Review Facility equity checklist

**S1: Search strategy**

We conducted electronic searches of the following 31 databases from inception, current to 7th March 2024:

***Built environment and transport:*** Transport Research International Documentation (TRID); ICE Virtual Library (Civil Engineering Resource); Geobase (OVID); Urbadoc; ICONDA (OVID)	***Health, and psychological and behavioural sciences***: CINAHL (EBSCO); Embase (OVID); MEDLINE (OVID); Public Health Database (Proquest); Health Management Information Consortium (OVID); PsycInfo (OVID); Cochrane Library - Databases of Systematic Reviews, and controlled trials (CENTRAL); Database of promoting health effectiveness reviews (DoPHER); The Trials Register of Promoting Health Interventions (TRoPHI)	***Social policy, social science and economics:*** Applied Social Sciences Index and Abstracts (ASSIA) (ProQuest); Econlit (EBSCO); ABI/Inform Global (Proquest); Sociological Abstracts (Proquest); Social Policy and Practice (OVID); PAIS Index (Proquest); ERIC (EBSCO); IBSS (ProQuest)	***Interdisciplinary, Web of Science:*** Science Citation Index (Web of Science); Social Science Citation Index (Web of Science); Conference Proceedings Citation Index - Science (Web of Science); Conference Proceedings Citation Index - Social Science & Humanities (Web of Science); Emerging Sources Citation Index (Web of Science)	***Other specialist resources:*** SafetyLit; Social Systems Evidence; Epistemonikos; Health Evidence Canada

Below we reproduce the MEDLINE search strategy and yields in full, with this MEDLINE search being adapted to the further 30 databases by the same information specialist.

MEDLINE search (conducted 7^th^ March 2024)

Database: Ovid MEDLINE(R) ALL <1946 to March 06, 2024>

1 ("active" adj2 ("commuting" or "commute" or "commutes" or "transportation" or "travel" or "traveling" or "travelling")).ti,ab,kf. (1993)

2 (("active" adj2 "transport") and (bicycl* or "bike" or "bikes" or "biking" or bikeability or cyclab* or "cycled" or "cycling" or cyclist* or handcycl* or monowheel* or wheelchair* or "skate" or "skating" or "skates" or skateboard* or rollerblad* or "roller blad*" or "e-bike" or "e-bikes" or "e-bikers" or "e-scooter*" or "e-skateboard*" or hoverboard* or skateboard* or scooter* or segway* or "Walk" or "walking" or "walked" or "walkability" or "walkable" or "wheeling" or pedestrian*)).ti,ab,kf. (437)

3 (("commuting" or "commute" or "commutes" or commuter* or "everyday journey" or "everyday journeys" or "utilitarian travel" or "utility travel" or "destination oriented" or "travel for a purpose" or "travelling for a purpose" or "traveling for a purpose" or ((travel* or transport* or journey*) adj2 ("school" or "schools" or "college" or "colleges" or "university" or "universities" or "workplace" or "workplaces" or "to work" or "for work" or "work place" or "worksite" or "worksites" or workforce* or "shops" or "shopping" or "all purpose" or "with a purpose" or "for a purpose" or purposeful)) or (((travel adj2 mode) or (travel adj2 modes) or (traveling adj2 mode) or (traveling adj2 modes) or (travelling adj2 mode) or (travelling adj2 modes) or (transport adj2 mode) or (transport adj2 modes) or (transportation adj2 mode) or (transportation adj2 modes)) and ("school" or "schools" or "college" or "colleges" or "university" or "universities" or "workplaces" or "to work" or "for work" or "work place" or "worksite" or workplace* or "work" or "worksite" or "worksites" or "workforce" or "shops" or "shopping"))) and ("physical activity" or "exercise" or "physically active" or (bicycl* or "bike" or "bikes" or "biking" or bikeability or cyclab* or "cycled" or "cycling" or cyclist* or handcycl* or monowheel* or wheelchair* or "skate" or "skating" or "skates" or skateboard* or rollerblad* or "roller blad*" or "e-bike" or "e-bikes" or "e-bikers" or "e-scooter*OR e-skateboard*" or hoverboard* or skateboard* or scooter* or segway* or "Walk" or "walking" or "walked" or "walkability" or "walkable" or "wheeling" or pedestrian*))).ti,ab,kf. (2443)

4 ("transport-related PA" or "transport-related exercis*" or ("transport*" adj2 (walk* or cycl* or bicycl* or "biking" or "wheeling" or "scootering"))).ti,ab,kf. (3094)

5 (("commuting" or "commute" or "commutes" or "transport" or "transportation" or "travel" or "traveling" or "travelling") and (bicycl* or "bike" or "bikes" or "biking" or bikeability or cyclab* or "cycling" or cyclist* or handcycl* or monowheel* or wheelchair* or skate or skating or skates or skateboard* or rollerblad* or "roller blad*" or "e-bike" or "e-bikes" or "e-bikers" or "e-scooter*OR e-skateboard*" or hoverboard* or skateboard* or scooter* or segway* or "Walk" or "walking" or "walked" or "walkability" or "walkable" or "wheeling" or pedestrian*) and ((behavio* adj2 change*) or (behavio* adj2 shift*) or (population* adj2 change*) or (population* adj2 shift*))).ti,ab,kf. (153)

6 (("physical activity" or "physically active") adj15 ("commuting" or "commute" or "commutes" or "transportation" or "travel" or "transport" or "traveling" or "travelling")).ti,ab,kf. (2305)

7 ((bicycl* or "bike" or "bikes" or "biking" or "bikeability" or cyclab* or "cycling" or cyclist* or handcycl* or monowheel* or wheelchair* or skate or skating or skates or skateboard* or rollerblad* or "roller blad*" or "e-bike" or "e-bikes" or "e-bikers" or "e-scooter*OR e-skateboard*" or hoverboard* or skateboard* or scooter* or segway* or "Walk" or "walking" or "walked" or "walkability" or "walkable" or "wheeling" or pedestrian*) adj15 "transport").ti,ab,kf. (2668)

8 ((bicycl* or "bike" or "bikes" or "biking" or bikeability or cyclab* or "cycling" or cyclist* or handcycl* or monowheel* or wheelchair* or skate or skating or skates or skateboard* or rollerblad* or "roller blad*" or "e-bike" or "e-bikes" or "e-bikers" or "e-scooter*" or "e-skateboard*" or hoverboard* or skateboard* or scooter* or segway* or "Walk" or "walking" or "walked" or "walkability" or "walkable" or "wheeling" or pedestrian*) adj15 "transportation").ti,ab,kf. (1509)

9 ((("e-bike" or "e-bikes" or "e-bikers" or "e-scooter" or "e-scooters" or "e-scootering" or "e-skateboard*" or hoverboard* or skateboard* or wheeling) adj2 ("school" or "schools" or "college" or "colleges" or "university" or "universities" or "workplaces" or "to work" or "for work" or "work place" or "worksite" or workplace* or "work" or "worksite" or "worksites" or "workforce" or "shops" or "utility" or "utilitarian" or destination* or "purpose" or "purposeful" or "trips")) or (("Walk" or "walking" or "walked" or walkabl* or "walkability" or "walkable" or pedestrian*) adj2 ("school" or "schools" or "college" or "colleges" or "university" or "universities" or "workplaces" or "to work" or "for work" or "work place" or "worksite" or workplace* or "work" or "worksite" or "worksites" or "workforce" or "shops" or "utility" or "utilitarian" or destination* or "purpose" or "purposeful" or "trips")) or ((bicycl* or "bike" or "bikes" or "biking" or "bikeability" or cyclab* or "cycling" or cyclist* or handcycl*) adj2 ("school" or "schools" or "college" or "colleges" or "university" or "universities" or "workplaces" or "to work" or "for work" or "work place" or "worksite" or workplace* or "work" or "worksite" or "worksites" or "workforce" or "shops" or "utility" or "utilitarian" or destination* or "purpose" or "purposeful" or "trips")) or ((monowheel* or wheelchair* or "skate" or "skating" or "skates" or skateboard* or rollerblad* or "roller blad*" or scooter* or segway*) adj2 ("school" or "schools" or "college" or "colleges" or "university" or "universities" or "workplaces" or "to work" or "for work" or "work place" or "worksite" or workplace* or "work" or "worksite" or "worksites" or "workforce" or "shops" or "utility" or "utilitarian" or destination* or "purpose" or "purposeful" or "trips"))).ti,ab,kf. (2316)

10 ((("salary sacrifice*" or (("work place" or workplace* or workforce* or worksite* or employe* or "staff" or "school" or commuter* or "commuting") adj8 (strategy* or "strategies" or "policy" or "policies" or "loan" or "loans" or subsid* or "grant" or "grants" or reward* or incentive* or intervention* or program* or scheme* or "campaign" or "campaigns" or initiative* or infrastructur* or prize*))) and (bicycl* or "bike" or "bikes" or "biking" or "bikeability" or cyclab* or "cycling" or cyclist* or handcycl* or monowheel* or wheelchair* or skate or skating or skates or skateboard* or rollerblad* or "roller blad*" or "e-bike" or "e-bikes" or "e-bikers" or "e-scooter*” OR e-skateboard*" or hoverboard* or skateboard* or scooter* or segway* or Walk or walking or walked or walkability or walkable or wheeling or pedestrian*)) or (("work place" or workplace* or workforce* or worksite* or employe* or "staff" or "school" or commuter* or "commuting") and ((strategy* or "strategies" or "policy" or "policies" or "loan" or "loans" or subsid* or "grant" or "grants" or reward* or incentive* or intervention* or program* or scheme* or "campaign" or "campaigns" or initiative* or infrastructur* or prize*) adj1 "cycle"))).ti,ab,kf. (1566)

11 (("Living Streets" or "Local Sustainable Travel Fund*" or LSTF or (("cycle" or "cycles" or "cycling" or cyclist* or "bike" or "bikes" or "biking" or "bikeway*" or bicycl* or walk* or pedestrian* or e-bike or e-scooter* or "wheeling" or scooter*) adj2 (superhighway* or "quietway" or "quietways" or "quiet way" or "quiet ways" or rides or maintenance or sharing or parking or share or park or hire or purchase or repair or refurbish* or recondition* or member* or grants or loan or loans or streetscap*)) or (("cycle" or "cycles" or "cycling" or cyclist* or "bike" or "bikes" or "biking" or "bikeway*" or bicycl* or walk* or pedestrian* or e-bike or e-scooter* or "wheeling" or scooter*) adj2 (networks or "led walks" or "led rides" or guided or "cycle rides" or "bicycle rides" or "bike rides")) or ("personalised travel planning" or "pavement improvement*" or "streetscape improvement*" or "pavement infrastructur*" or "streetscape infrastructur*" or "cycle routes" or "walking routes" or "pedestrian routes" or "cycle superhighway*" or cyclescheme* or "Cycling Cities" or "Cycling Towns" or "Cycle City" or "Cycling Demonstration Towns" or "cycleway*" or "bikeway*" or "cycle way*" or "bike way*") or (("cycle" or "cycles" or "cycling" or cyclist* or "bike" or "bikes" or "biking" or "bikeway*" or bicycl* or walk* or pedestrian* or e-bike or e-scooter* or "wheeling" or scooter*) adj5 (strategy* or strategies or policy or policies or program* or initiative* or loan or loans or subsid* or grant or grants or reward* or incentive* or intervention* or program* or scheme* or campaign or campaigns or initiative* or infrastructur* or prize* or training or "Smarter Choices" or lane or lanes or routes or route or festivals or events or training or classes or paths or pathways or promot* or inclusion or "Links to Schools")) or ("walking bus" or "walking buses" or "Bicycle Trains" or "bicycle train" or travelsmart or "safe routes to school" or "safe routes to schools" or "beat the streets" or "walk in to work out" or "Bike It" or "Making Travel Plans Work" or "Walk to School" or "Walking Once a Week")) and ("commuting" or "commute" or "commutes" or commuter* or "everyday journey" or "everyday journeys" or "utilitarian travel" or "utility travel" or "destination oriented" or "travel for a purpose" or "travelling for a purpose" or "traveling for a purpose" or ((travel* or transport* or journey*) adj2 ("school" or "schools" or "college" or "colleges" or "university" or "universities" or "workplace" or "workplaces" or "to work" or "for work" or "work place" or "worksite" or "worksites" or workforce* or "shops" or "shopping" or "all purpose" or "with a purpose" or "for a purpose" or purposeful)) or (((travel adj2 mode) or (travel adj2 modes) or (traveling adj2 mode) or (traveling adj2 modes) or (travelling adj2 mode) or (travelling adj2 modes) or (transport adj2 mode) or (transport adj2 modes) or (transportation adj2 mode) or (transportation adj2 modes)) and ("school" or "schools" or "college" or "colleges" or "university" or "universities" or "workplaces" or "to work" or "for work" or "work place" or "worksite" or workplace* or "work" or "worksite" or "worksites" or "workforce" or "shops" or "shopping")))).ti,ab,kf. (512)

12 (("active" adj2 "transport") and ("commuting" or "commute" or "commutes" or commuter* or "everyday journey" or "everyday journeys" or "utilitarian travel" or "utility travel" or "destination oriented" or "travel for a purpose" or "travelling for a purpose" or "traveling for a purpose" or ((travel* or transport* or journey*) adj2 ("school" or "schools" or "college" or "colleges" or "university" or "universities" or "workplace" or "workplaces" or "to work" or "for work" or "work place" or "worksite" or "worksites" or workforce* or "shops" or "shopping" or "all purpose" or "with a purpose" or "for a purpose" or purposeful)))).ti,ab,kf. (261)

13 ("active" adj2 "transport").ti,ab,kf. and (exercise/ or exp walking/ or exp Bicycling/ or Built environment/ or Urban planning/ or Transportation/ or exp Population Health/ or Health Promotion/ or Health Education/ or Health Behavior/ or Schools/ or Public Health/ or Work/ or schools, nursery/ or universities/ or Environment Design/) (531)

14 Transportation/ and (exercise/ or exp walking/ or exp Bicycling/ or exp Population Health/ or Health Promotion/ or Health Education/ or Health Behavior/ or Schools/ or Public Health/ or Work/ or schools, nursery/ or universities/) (2444)

15 1 or 2 or 3 or 4 or 5 or 6 or 7 or 8 or 9 or 10 or 11 or 12 or 13 or 14 (13409)

16 (((review or synthes*) adj5 ("evidence" or intervention*)) or (weight adj2 evidence) or ("intervention study" or efficac* or "effects" or "impact" or effective* or evaluat* or "feasibility study" or "meta-analysis" or "meta-analyse*" or postintervention or "post intervention" or "pre intervention" or "preintervention" or (controlled adj2 design) or "pilot study" or "pilot studies" or "pilot program*" or "pilot project" or "feasibility studies" or "pilot studies" or "outcome*" or "performance measure*" or "performance assessment*" or "mixed methods" or "proof of concept" or longitudinal or "cost benefit" or "cost-effective" or (program* adj2 effect*) or (program* adj2 implementation) or (mechanism* adj2 change) or "observed effects" or "observed effect*" or "RCT" or "randomized" or "randomised" or "trial" or "trials" or cRCT or "cluster-random*" or "experiment" or "experiments" or "experimental" or "Quasi-experimental" or "stage of change" or "stages of change") or ("randomly" or ((random* or "controlled" or "parallel" or "crossover" or "cross over" or "blind" or "comparison" or "comparative") adj2 ("studies" or "study" or "design" or "trial" or "trials" or group*)) or (open adj1 label) or ((double or single or doubly or singly) adj1 (blind or blinded or blindly)) or "control group")).ti,ab,kf. or ("evidence" or "effect").ti. or ((review or synthesis or evidence or reviews) and (strategy* or "strategies" or "policy" or "policies" or "loan" or "loans" or subsid* or "grant" or "grants" or reward* or incentive* or intervention* or program* or scheme* or "campaign" or "campaigns" or initiative* or infrastructur* or prize*)).ti,kf. (14803187)

17 feasibility studies/ or pilot projects/ or program evaluation/ (289267)

18 clinical study/ or clinical trial/ or adaptive clinical trial/ or clinical trial, phase i/ or clinical trial, phase ii/ or clinical trial, phase iii/ or clinical trial, phase iv/ or controlled clinical trial/ or randomized controlled trial/ or equivalence trial/ or pragmatic clinical trial/ or comparative study/ or evaluation study/ or meta-analysis/ or multicenter study/ or "systematic review"/ (3396833)

19 controlled before-after studies/ or historically controlled study/ or interrupted time series analysis/ (2959)

20 16 or 17 or 18 or 19 (15950259)

21 15 and 20 (7951)

22 (exp animal/ or exp invertebrate/ or animal experiment/ or animal model/ or exp plant/ or exp fungus/) not exp human/ (5637348)

23 21 not 22 (7469)

-------------------------------------------------------------------------------------------------------------------

Our searches originally targeted a wider literature than that focused on in the current systematic review and therefore did not include terms to attempt to limit their scope in key aspects relevant to the current review, such as limiting searches to specify primary research conducted in the UK. This was because we were initially commissioned by the commissioners of this review, the Department of Health and Social Care (England), to map the existing literature on the equity impacts of active travel interventions including both primary and secondary research (i.e. systematic reviews and other evidence syntheses) and with an international, not solely UK, focus (Kwan et al, in preparation). This phase of work then informed their subsequent commissioning of the current systematic review that more narrowly-focused only on primary research conducted in the UK. Screening conducted for the prior broad mapping work meant we were able to identify relevant primary research reports evaluating active travel interventions, which were re-screened for this review and supplemented by searching reference lists of included reports, relevant systematic reviews (these having also been identified in the prior broader mapping work) and contacting authors of included studies.

**S2: Description of evaluated interventions**

Following TIDieR guidance (Hoffmann et al, 2014), below we describe the interventions using a TIDieR table and narrative summary.

**Table. Details of five active travel interventions in the UK: TIDieR-PHP (Template for Intervention Description and Replication for Public Health and Policy)**

| **Intervention** | **How was it theorised to work?** | **What materials did the intervention comprise?** | **How was the intervention planned?** | **Who commissioned / delivered it?** | **Where and when was it implemented?** |
| --- | --- | --- | --- | --- | --- |
| **Name:** Cambridgeshire Guided Busway  **Primary report used:**  Panter et al, (2016)  **BCT:** Antecedents: (Restructuring the physical environment/ Adding objects to the environment) | ‘..,exposure to new infrastructure to promote walking, cycling, and public transport—the Cambridgeshire Guided Busway —would result in an increase in time spent walking and cycling on the commute and higher levels of overall physical activity’ (Panter et al, 2016 p.e46) | **Cost:** Approx £152M (h[ttps://en.wikipedia.org/wiki/Cambridgeshire_Guided_Busway](https://en.wikipedia.org/wiki/Cambridgeshire_Guided_Busway))  **Physical infrastructure:** A new bus network and an adjacent 22-kilometer a traffic-free walking and cycling route. (Panter et al, 2016 p.e46)  **Details:** Buses run on a guideway completely segregated from other traffic along much of the route, and a new high-quality surfaced path for pedestrians and cyclists runs alongside. Park-and-ride interchange facilities.  **Other intervention elements:** None | Proposed in 2001 Cambridge-Huntingdon Multi-Modal Study, which recommended widening the A14 road and the construction of a guided busway along the old railway lines. | Commissioned and delivered by Cambridgeshire County Council (<https://en.wikipedia.org/wiki/Cambridgeshire_Guided_Busway>)  Bus services operated by the Stagecoach Group and Whippet Coaches | **Where:**  Cambridge  **When:**  2011 |
| **Name:** Cycling Towns  **Primary report used:**  Patterson et al, (2023); Sloman et al, (2009).  **BCT:** Antecedents: (Restructuring the physical environment/ Adding objects to the environment). Plus site-specific initiatives (see ‘Use of Behaviour Change Techniques’ in Supplementary Material S2).  See also: https://ars.els-cdn.com/content/image/1-s2.0-S0277953613004826-mmc3.doc | '...a mixture of capital investment (e.g. building cycle lanes, creating cycle parking) and revenue investment (e.g. promotional activities, cycle training) would impact on the prevalence of travelling to work by bicycle and other modes' (Goodman et al, 2013 p 229). | **Cost:** CDTs: £15m; CCTs: £50m ([www.nice.org.uk/guidance/ng246/evidence/ph42-expert-paper-6-lingwood-pdf-430268083](https://www.nice.org.uk/guidance/ng246/evidence/ph42-expert-paper-6-lingwood-pdf-430268083)); (www.cycling-embassy.org.uk/sites/cycling-embassy.org.uk/files/documents/cct-qualitative-research.pdf)  **Physical infrastructure:** Cycle lanes and tracks in 6 Cycling Demonstration Towns (CDTs) and 12 Cycling Cities and Towns (CCTs).  **Details:** 'More than 360 km of cycle lanes and tracks [...] an increase of more than 30% over the study period. Measures such as traffic calming, speed restriction and improved junctions also added in many areas. Neighbourhood events, workplace support for journey planning, and school-based cycle training (Bikeability) were also provided.' (Patterson et al, 2023 p2).  **Other intervention elements:** Behavioural components | Local authorities of towns were supported by Cycling England in planning through strategic and specialist technical advice on cycling infrastructure. The towns worked closely together with other CCTs and to learn from the earlier experience of the CDTs, which had been implementing programmes to increase cycling since 2005.  (www.sustrans.org.uk/media/2977/2977.pdf) | A combination of central government funding (Cycling England / DfT / DH) and matched local authority funding. | **Where:**  CDTs: Darlington, Lancaster, Exeter, Aylesbury, Derby and Brighton and Hove.  CCTs: Blackpool, Bristol, Cambridge, Chester, Colchester, Leighton/Linslade, Shrewsbury, Southend-on-Sea, Southport with Ainsdale, Stoke, Woking and York  **When:**  CDTs: 2005; CCTs: 2008 |
| **Name:** ENABLE London  **Primary report used:**  Owen et al, (2020); Limb et al, (2020)  **BCT:** Antecedents: (Restructuring the physical environment/ Adding objects to the environment) | '.....those living in social, intermediate and market-rent accommodation in East Village show a sustained change in their physical activity levels, compared with their levels before moving into the Village, and compared with physical activity change among those who did not move into East Village? (Owen et al, 2020 p3) | **Cost:** £180M (https://www.architectsjournal.co.uk/buildings/hawkinsbrown-completes-180m-stratford-east-village-housing-scheme)  **Physical structure:**  A purpose-built mixed-use residential development specifically designed to encourage healthy active living.  **Details:** East Village, part of the regeneration of the former 2012 London Olympic site, was repurposed/redesigned to provide housing with high levels of walkability and proximity to open parkland and improved public transport. It consists of 3 different housing types: 1. Social housing – approximately 675 units. 2. Intermediate accommodation - 704 units. 3. Market-rent accommodation – 1439 units. (Owen et al, 2020 p3).  **Other intervention elements:** None | Planning involved close collaboration with various stakeholders (investors Qatari Diar and Delancey, architects Grant Associates, rental operator Get Living London) approved by the London Legacy Development Corporation (LLDC). | The East Village project was commissioned by the Olympic Delivery Authority (ODA). The development is a joint venture between Qatari Diar and Delancey, Triathlon Homes, First Base, East Thames Group and Southern Housing Group. | **Where:**  Stratford, East London.  **When:**  Planned opening: 2013; Actual opening: 2014 |
| **Name:** iConnect*/Connect2**  **Primary report used:**  Goodman et al, (2014); Panter & Ogilvie (2017); Song et al, (2017)  **BCT**: Antecedents: (Restructuring the physical environment/ Adding objects to the environment)  * The iConnect study, aims to measure and evaluate the travel, physical activity and carbon impacts of the Connect2 program. (http://www.iconnect.ac.uk)**Connect2 is a program led by Sustrans to build or improve local walking and cycling routes around the UK. (http://www.sustransconnect2. org.uk) | '...living closer to the new walking and cycling infrastructure (traffic-free bridges and boardwalk) would lead to increases in walking, cycling and overall physical activity' (Panter & Ogilvie, 2017 p529) | **Cost:** £175M† (le Gouais 2021) ; Connect2 Cardiff: £4.6M; Connect2 Kenilworth: £1M; Connect2 Southampton: £1M; (Ogilvie et al, 2012, p5; Goodman et al, 2014 supp materials)  **Physical infrastructure:** Traffic-free pedestrian/cyclist bridges and boardwalk.  **Details:** (1) Cardiff, a traffic-free bridge over Cardiff Bay, connecting Penarth to the city; (2) Kenilworth, a traffic-free walking and cycling bridge over a busy trunk road; and (3) Southampton, an informal riverside footpath turned into a raised boardwalk linking city centre and nearby residential areas along River Itchen.  **Other intervention elements:** None  †for all 84 Connect2 walking and cycling infrastructure programs across the UK | Led by Sustrans working with dozens of stakeholders, including local government, statutory and non-statutory bodies and local community groups, to deliver 84 networks of walking and cycling routes. | Supported by a grant from the Big Lottery Fund (£50M), the Connect 2 programme completed many high-quality walking and cycling routes between 2009 and 2013 | **Where:** Cardiff; Kenilworth; Southampton  **When:** Cardiff and Southampton, July 2010; Kenilworth, September 2011 |
| **Name:** Mini-Hollands  **Primary report used:**  Aldred et al, (2019)  **BCT:** Antecedents: (Restructuring the physical environment/ Adding objects to the environment) | '...creating pedestrian and cycling-friendly street environments in supporting a shift from private car use to active transport modes' (Aldred et al, 2019 p149) | **Costs:** £100M (p149)  **Physical infrastructure:** The infrastructure changes include redesigned town centres, with cycle hubs at tube and rail stations; measures to reduce and calm motor traffic in residential areas; and physically protected cycle lanes along main roads.  **Details:** This included separate bus and cycle lanes, a Dutch-style roundabout, cycle Hubs, cycling bridge and cycle path network, traffic calming, ‘Copenhagen crossings’, segregated cycle lanes'. (<https://en.wikipedia.org/wiki/Mini-Hollands>); (https://www.london.gov.uk/programmes-strategies/transport/cycling-and-walking/transforming-cycling-outer-boroughs-mini-hollands-programme) | Planning by 'Transport for London (TfL) and the 3 mini-Holland boroughs (https://www.gov.uk/government/case-studies/london-mini-hollands). | The scheme was funded by TfL as part of a commitment that the Mayor of London (2008–16), Boris Johnson, made to better protect vulnerable road users, learning from Dutch cycling provision (Di and Palmieri, 2016).  Each of the 3 participating boroughs was awarded £30M funding to transform the environment for cycling.  The programme has now evolved to be part of the more holistic Healthy Streets approach (TfL, 2017b). | **Where:** London Boroughs of Enfield, Waltham Forrest, Kingston  **When:** 2021 |

BCT= Behavior Change Techniques; EBABLE = Examining Neighbourhood Activities in Built Living Environments in London; iConnect = Impact of Constructing Non-motorised Networks and Evaluating Changes in Travel;

**Narrative summary**

Brief details of the interventions are presented using the TIDierR –PHP (Template for Intervention Description and Replication for Public Health and Policy) (Hoffmann 2014; Campbell 2018) which is an extension of the CONSORT 2010 (item 5) checklist (Schulz 2010).

The five intervention programmes in England encompassed the restructuring of the physical environment to promote walking and cycling for active travel in a variety of ways.

The Cambridgeshire Guided Busway (CGB) is a bus guideway of concrete track with an adjacent 22-kilometer a traffic-free walking and cycling route in Cambridge. Proposed in 2001 and commissioned by the Cambridgeshire County Council, it was implemented in 2011 and operated by the Stagecoach Group and Whippet Bus Company. Estimated cost: £152M.

The Cycling Towns programme involved 6 Cycling Demonstration Towns (CDTs: Darlington, Lancaster, Exeter, Aylesbury, Derby and, Brighton and Hove) in 2005 and subsequently 12 Cycling Towns and Cities (CTTs: Blackpool, Bristol, Cambridge, Chester, Colchester, Leighton/Linslade, Shrewsbury, Southend-on-Sea, Southport with Ainsdale, Stoke, Woking and York) in 2008. Over 360 km of cycle lanes and tracks was constructed with additional measures of traffic calming, speed restriction and improved junctions. It was supported by a combination of central government funding (Cycling England / Department for Transport / Department of Health) and matched local authority funding. Part of the intervention included a behavioural element such as school-based cycle training. Estimated cost: £15M for CDTs; £50M for CCTs.

The ENABLE report evaluated East Village (Stratford, East London) which, as part of the regeneration of the former 2012 London Olympic site, was repurposed/redesigned to provide housing with high levels of walkability and proximity to open parkland and improved public transport. It consists of three different housing types: Social housing, Intermediate accommodation and Market-rent accommodation. It was commissioned by the Olympic Delivery Authority (ODA) and opened in 2014. The development is a joint venture between Qatari Diar and Delancey, Triathlon Homes, First Base, East Thames Group and Southern Housing Group. Estimated cost: £180M.

The iConnect report evaluated three sites from the Connect2 programme which was led by the transport charity Sustrans to build or improve access to walking and cycling for communities across the UK. The three study sites were: Cardiff where a traffic-free bridge was built over Cardiff Bay; Kenilworth, a traffic-free bridge over a busy trunk road; and Southampton where an informal riverside footpath was turned into a boardwalk. These infrastructures were implemented between 2010 and 2011. Estimated cost: £4.6M in Cardiff; £1M in Kenilworth; £1M in Southampton. Connect2 was supported by a grant from the Big Lottery Fund and 84 walking and cycling routes have since been created or upgraded across the UK. Estimated cost of the Connect2 programme: £175M.

The Mini-Hollands scheme was part of the London Mayor’s Healthy Streets Approach (2008-16) aiming specifically at outer London boroughs where residents are more car-dependent than inner London. Infra-structures such as cycle hub, bridge and traffic calming were created to be as pedestrian and cycle-friendly as the Dutch-equivalents in three London boroughs: Enfield, Walthamstow and Kingston. The scheme, implemented in 2021, was funded by Transport for London (Tfl) and formed part its Vision for Cycling 2013. Each of the three participating boroughs was awarded £30 million funding to transform the environment for cycling. Estimated cost: £100M.

**References:**

Campbell M, Katikireddi SV, Hoffmann T, Armstrong R, Waters E, Craig P. TIDieR-PHP: a reporting guideline for population health and policy interventions. BMJ. 2018;361:k1079;

Hoffmann TC, Glasziou PP, Boutron I, et al. Better reporting of interventions: template for intervention description and replication (TIDieR) checklist and guide. BMJ 2014;348:g1687. doi:10.1136/ bmj.g1687;

Le Gouais, Anna, et al. "A natural experimental study of new walking and cycling infrastructure across the United Kingdom: The Connect2 programme." *Journal of Transport & Health* 20 (2021). <https://doi.org/10.1016/j.jth.2020.100968>

Ogilvie D, Bull F, Cooper A, et al. Evaluating the travel, physical activity and carbon impacts of a ‘natural experiment’ in the provision of new walking and cycling infrastructure: methods for the core module of the iConnect study. BMJ Open 2012;2:e000694. doi:10.1136/ bmjopen-2011-000694

Schulz KF, Altman DG, Moher DCONSORT Group. CONSORT 2010 statement: updated guidelines for reporting parallel group randomised trials. BMJ 2010;340:c332. doi:10.1136/bmj.c332.

**Use of Behaviour Change Techniques**

**Summary**

All the included interventions involved restructuring the physical environment and adding objects to the environment (BCT 12: Antecedents). One study also incorporated a variety of site-specific behavioural change techniques (Cycling Towns; see Table below for details). However these varied between the 18 towns included within this study and it was not possible to establish which combinations of techniques are most effective.

**Table: Behaviour Change Techniques (BCT)* reported in the included interventions**

| **Interventions** | **BCT activities** | | | | | | | | | | |  |
| --- | --- | --- | --- | --- | --- | --- | --- | --- | --- | --- | --- | --- |
|  | ***12. Antecedents: (Restructuring the physical environment /* *Adding objects to the environment)*** | ***1. Goals and planning*** | ***3. Social support*** | ***4. Shaping knowledge*** | ***5. Natural consequences*** | ***6. Comparison of behaviour /* *outcomes*** | ***7. Associations*** | ***8. Repetition and substitution*** | ***10. Reward and threat*** | ***14. Scheduled consequences*** | ***Notes*** |  |
| **Cambridge shire Guided Busway** | **√** | **-** | **-** | **-** | **-** | **-** | **-** | **-** | **-** | **-** | **--** |  |
| ****Cycling Towns (18 sites)** | **√** | **√** | **√** | **√** | **√** | **√** | **√** | **√** | **√** | **√** | Activities varied by site – not all BCTs were in all sites |  |
| **ENABLE London** | **√** | **-** | **-** | **-** | **-** | **-** | **-** | **-** | **-** | **-** | **--** |  |
| **iConnect** | **√** | **-** | **-** | **-** | **-** | **-** | **-** | **-** | **-** | **-** | **--** |  |
| **Mini-Hollands** | **√** | **-** | **-** | **-** | **-** | **-** | **-** | **-** | **-** | **-** | **--** |  |

n/r=not reported. Sources: *Michie 2013: BCTT v1: <https://doi.org/10.1007/s12160-013-9486-6>. **Goodman 2013 (Supp 1): <https://ars.els-cdn.com/content/image/1-s2.0-S0277953613004826-mmc3.doc> (NB: the article and supplementary material contain brief summaries of intervention activities. As such some categorisation may be incomplete or incorrect). Not all activities or BCTs were present in all 18 sites.

**S3: Risk of bias assessment**

As outlined in the main text, we independently assessed risk of bias (termed ‘quality assessment in the EPHPP tool) before reaching agreement by consensus. We conducted six risk of bias (framed as ‘quality’ by the EPHPP tool) assessments, since one intervention (Cycling Towns) had two distinct evaluations that used different methods and data sources (Patterson 2023; Sloman 2009). One evaluation was rated as strong overall (Cycling Towns (Patterson 2023)), one as moderate (ENABLE London), and four as weak (Cambridgeshire Guided Busway; Cycling Towns (Sloman 2009); iConnect; Mini-Hollands). Three of the four weak evaluations had three domains rated weak; the fourth had two weak domains. The weakest domain across evaluations overall was withdrawals and dropouts, for which all but one evaluation was rated weak and none as strong. The strongest domain was blinding, for which no evaluations were rated as weak and one was considered strong (Cycling Towns (Patterson 2014)) followed by study design, where one evaluation (Cycling Towns (Sloman 2009)) was rated weak and the remaining as moderate (with none rated strong, since none were randomised controlled trials).

**Table: Quality assessment of studies included in the review, using the EPHPP Quality Assessment Tool for Quantitative Studies**

| **Intervention name** | **Evaluation reports used** | **A Selection Bias** | **B Study Design** | **C Confounders** | **D Blinding** | **E Data Collection Methods** | **F Withdrawals and Dropouts** | **Overall rating** |
| --- | --- | --- | --- | --- | --- | --- | --- | --- |
| **Cambridgeshire Guided Busway** | Panter et al, (2016) | Weak | Moderate | Strong | Moderate | Strong | Weak | **Weak** |
| **Cycling Towns** | Patterson et al, (2023)* | Strong | Moderate | Strong | Strong | Strong | Moderate | **Strong** |
|  | Sloman et al, (2009)* | Moderate | Weak | Strong | Moderate | Weak | Weak | **Weak** |
| **ENABLE** | Limb et al, (2020)  Owen et al, (2020) | Strong | Moderate | Moderate | Moderate | Strong | Weak | **Moderate** |
| **iConnect** | Goodman et al, (2014)  Panter & Ogilvie, (2017)  Song (2017) | Weak | Moderate | Strong | Moderate | Weak | Weak | **Weak** |
| **Mini-Hollands** | Aldred et al, (2019) | Weak | Moderate | Weak | Moderate | Moderate | Weak | **Weak** |

*Although Patterson et al, (2023) and Sloman et al, (2009) both evaluated the Cycling Towns intervention, they conducted different analyses using different methods and data sources. Therefore, we conducted separate risk of bias assessments for each.

**S4: Data underlying effect direction plot (Figure 2 in main paper)** *Green highlighting indicates statistical significance of effect*

Sex/gender (female vs male)

|  | **Outcome** | **Analysis** | **Direction of intervention effect *within* less advantaged population subgroup(s)** | **Direction of effect in less advantaged subgroup(s) *relative to* more advantaged subgroup(s)** | **Data source** |
| --- | --- | --- | --- | --- | --- |
| 1. **CAMBRIDGE GUIDED BUSWAY** |  |  |  |  |  |
| **Active travel** | **Weekly time (in minutes) spent in active commuting (categorised as increase, no change, or decrease**) | Regression analyses testing intervention effects at three years post-baseline and interactions to examine whether these effects varied by participant characteristics | **RR (95% CI)**    **Female** Increased 1.27 (0.94 – 1.71) Decreased 1.15 (0.83 - 1.59)  **Male** Increased 0.98 (0.60 – 1.59) Decreased 0.88 (0.55 – 1.42)    **↔** *Mix of positive and negative effects for females; positive effect on increased active commuting (AC) but also on decreased AC (both non-significant)* | **RR (95% CI)**    **Female** Increased 1.27 (0.94 – 1.71) Decreased 1.15 (0.83 -1.59)  **Male** Increased 0.98 (0.60 – 1.59) Decreased 0.88 (0.55 – 1.42)    **↔** *Mix of greater and lesser effects (greater effect on increased AC in females than males, but also greater effect on decreased AC in females than males)* | Panter et al (2016) plus regression data direct from Authors |
| 2. **CYCLING TOWNS** |  |  |  |  |  |
| **Walking** | **Walking as usual commute mode** | Difference-in-differences (DID) between 2001 and 2011 intervention towns vs matched comparison towns | **AOR (95% CI)**    **Female** 1.24 (1.08 - 1.43)  **Male** 1.08 (0.90 - 1.30)    **↑** *Positive effect for females (significant)* | **AOR (95% CI**)    **Female** 1.24 (1.08 - 1.43)  **Male** 1.08 (0.90 - 1.30)    **↑** *Greater effect in females than males (non-significant)* | Patterson et al (2023) Supplemental Table 7 |
| **Cycling** | **Cycling as usual commute mode** | DID estimate between 2001 and 2011 intervention towns vs matched comparison towns | **AOR (95% CI)**    **Female** 1.56 (1.16 - 2.10)  **Male** 0.91 (0.76 - 1.10)    **↑** *Positive effect for females (significant)* | **AOR (95% CI)**    **Female** 1.56 (1.16 - 2.10)  **Male** 0.91 (0.76 - 1.10)    **↑** *Greater effect in females than males (significant)* | Patterson et al (2023) Supplemental Table 7 |
|  | **Taking up cycling** | Association between living in an intervention area and taking up cycling in 2011 compared with matched control group | **AOR (95% CI)**    **Female** 2.13 (1.56 - 2.91) p=<0.001  **Male** 1.19 (0.93 - 1.51) p=0.164    **↑** *Positive effect for females (significant)* | **AOR (95% CI)**    **Female** 2.13 (1.56 - 2.91)  **Male** 1.19 (0.93 - 1.51)    **↑** *Greater effect in females than males (significant)* | Patterson et al (2023) Supplemental Table 8 |
|  | **Maintaining cycling** | Association between living in an intervention area and taking up cycling in 2011 compared with matched control group | **AOR (95% CI)**    **Female** 1.65 (0.91 - 2.98) p=0.099  **Male** 1.06 (0.76 - 1.50) p=0.722    **↑** *Positive effect for females (non- significant)* | **AOR (95% CI)**    **Female** 1.65 (0.91 - 2.98)  **Male** 1.06 (0.76 - 1.50)    **↑** *Greater effect in females than males (non-significant)* | Patterson et al (2023) Supplemental Table 8 |
| **Active travel** | **Cycling or walking as usual commute mode** | DID estimate between 2001 and 2011 intervention towns vs matched comparison towns | **AOR (95% CI)**    **Female** 1.32 (1.16 - 1.51)  **Male** 1.00 (0.87 - 1.14)    **↑** *Positive effect for females (significant)* | **AOR (95% CI)**    **Female** 1.32 (1.16 - 1.51)  **Male** 1.00 (0.87 - 1.14)    **↑** *Greater effect in females than males (significant)* | Patterson et al (2023) Supplemental Table 7 |
| 3. **ENABLE LONDON** |  |  |  |  |  |
|  | Not available | Not available | Not available | Not available | Not available |
| 4. **iCONNECT** |  |  |  |  |  |
| **Walking** | **Change in walking for transport** | Regression analyses testing intervention effects at two years and interactions to examine whether these effects varied by participant characteristics | **Coefficient (B) from maximally adjusted model (95% CI)**    **Female** 8.3 [-2.3 - 18.9] p=0.124  **Male** 7.8 [-3.5 - 19.1] p=0.176    **↑** *Positive effect for females (non-significant)* | **Coefficient (B) from maximally adjusted model (95% CI)**    **Female** 8.3 [-2.3 - 18.9]  **Male** 7.8 [-3.5 - 19.1]  Interaction p=0.994    **↑** *Greater effect in females than males (non-significant)* | Goodman et al (2014) plus regression data direct from Authors |
|  | **Short-lived increase in walking for transport** | Latent class analysis (LCA) to identify groups whose walking changed in similar ways and multinomial regression to examine factors associated with group membership | Not available | **RRR (95% CI)**    **Female** 1.0 (Ref. category)  **Male** 0.76 (0.38 - 1.49)    **↑** *Greater effect in females than males (non- significant)* | Panter et al (2017) Table 4 |
|  | **Sustained increase in walking for transport** | As above | Not available | **RRR (95% CI)**    **Female** 1.0 (Ref. category)  **Male** 0.95 (0.53 - 1.71)    **↑** *Greater effect in females than males (non- significant)* | Panter et al (2017) Table 4 |
|  | **Uptake of walking for transport** | As above | Not available | **OR (95% CI)**    **Female** 1.0 (Ref. category)  **Male** 0.89 (0.47 - 1.72)    **↑** *Greater effect in females than males (non-significant)* | Panter et al (2017) Table 4 |
| **Cycling** | **Change in cycling for transport** | Regression analyses testing intervention effects at two years and interactions to examine whether these effects varied by participant characteristics | **Coefficient (B) from maximally adjusted model (95% CI**)    **Female** 1.1 [-1.0 – 3.1] p=0.308  **Male** -2.8 [-5.7 – 0.1] p= 0.060    **↑** *Positive effect for females (non-significant)* | **Coefficient (B) from maximally adjusted model (95% CI)**    **Female** 1.1 [-1.0 – 3.1]  **Male** -2.8 [-5.7 – 0.1]  Interaction p=0.104    **↑** *Greater effect in females than males (non-significant)* | Goodman et al (2014) plus regression data direct from Authors |
| **Active travel** | **Modal shift from private motor vehicles to walking and cycling after two years in terms of time share** | Logistic regression modelling associations with modal shift in terms of *time* after two years, with distance to core C2 as measure of exposure | Not available | **Coefficients (t-value)**    **Female** -0.29 (-3.55)  **Male** (Ref. category)  p=<0.01    **↓** *Lesser effect in females than males (significant)* | Song et al (2017) Table 6 |
|  | **Modal shift from private motor vehicles to walking and cycling after two years in terms of distance share** | Logistic regression modelling associations with modal shift in terms of *distance*, with distance to core C2 as measure of exposure | Not available | **Coefficients (t-value)**    **Female** -0.40 (4.67)  **Male** (Ref. category)  p=<0.01    **↓** *Lesser effect in females than males (significant)* | Song et al (2017) Table 6 |
| 5. **MINI HOLLANDS** |  |  |  |  |  |
| **Cycling** | **Past week cycling minutes** | Regression analyses testing for interactions to examine whether intervention effects after three years varied according to participant characteristics | Not available | Direction of effect data provided to review team by authors    **↑** *Greater effect in females than males (non-significant)* | Aldred et al (2019) plus data from Authors |
| **Active travel** | **Past week active travel minutes** | As above | Not available | Direction of effect data provided to review team by authors    **↓** *Lesser effect in females than males (non-significant)* | Aldred et al (2019) plus data from Authors |

Age (older vs youngest adults)

|  | **Outcome** | **Analysis** | **Direction of intervention effect *within* less advantaged population subgroup(s)** | **Direction of effect in less advantaged subgroup(s) *relative to* more advantaged subgroup(s)** | **Data source** |
| --- | --- | --- | --- | --- | --- |
| 1. **CAMBRIDGE GUIDED BUSWAY** |  |  |  |  |  |
| **Active travel** | **Weekly time (in minutes) spent in active commuting**  **(categorised as increase no change, or decrease**) | Regression analyses testing intervention effects at three years post-baseline and interactions to examine whether these effects varied by participant characteristics | **RR (95% CI)**    **> 50 yrs** Increased 0.76 (0.47 – 1.23) Decreased 1.04 (0.71 – 1.53)  **40-50 yrs** Increased 1.90 (1.09 – 3.31) Decreased 1.07 (0.59 – 1.92)  **< 40 yrs** Increased 1.16 (0.68 – 1.99 Decreased 1.17 (0.66 – 2.06)    **↔** *Mixed effects: for >50yrs negative effects (lesser effect on increased AC and greater effect on decreased AC, both non-significant);  40-50yrs mixed effects (greater effect on increased AC, significant, and greater effect on decreased AC, non-significant).* | **RR (95% CI)**    **> 50 yrs** Increased 0.76 (0.47 – 1.23) Decreased 1.04 (0.71 – 1.53)  **40-50 yrs** Increased 1.90 (1.09 – 3.31) Decreased 1.07 (0.59 – 1.92)  **< 40 yrs** Increased 1.16 (0.68 – 1.99 Decreased 1.17 (0.66 – 2.06)    **↔** *Mix of greater and lesser effects in older two age groups than <40 yrs* (non-significant) | Panter et al (2016) plus regression data direct from Authors |
| 2. **CYCLING TOWNS** |  |  |  |  |  |
| **Walking** | **Walking as usual commute mode** | Difference-in-differences (DID) between 2001 and c2011 intervention towns vs matched comparison towns | **AOR (95% CI)**    **40+ yrs** 1.18 (1.01 - 1.38)  **16-39 yrs** 1.19 (1.02 - 1.38)    **↑** *Positive effect for older age group (significant)* | **AOR (95% CI)**    **40+ yrs** 1.18 (1.01 - 1.38)  **16-39 yrs** 1.19 (1.02 - 1.38)    **↔** *Same effect in older as in younger age group [to one decimal place] (non-significant)* | Patterson et al (2023) Supplemental Table 7 |
| **Cycling** | **Cycling as usual commute mode** | DID estimate between 2001 and 2011 intervention towns vs matched comparison towns | **AOR (95% CI)**    **40+ yrs** 1.27 (1.01 - 1.61)  **16-39 yrs** 0.97 (0.78 - 1.20)    **↑** *Positive effect for older age group (significant)* | **AOR (95% CI)**    **40+ yrs** 1.27 (1.01 - 1.61)  **16-39 yrs** 0.97 (0.78 - 1.20)    **↑** *Greater effect in older than younger age group (non-significant)* | Patterson et al (2023) Supplemental Table 7 |
|  | **Taking up cycling** | Association between living in an intervention area and taking up cycling in 2011 compared with matched control group | **AOR (95% CI)**    **40+ yrs** 1.95 (1.42 - 2.69) p=<0.001  **16-39 yrs** 1.27 (1.01 - 1.61) p=0.044    **↑** *Positive effect for older age group (significant)* | **AOR (95% CI)**    **40+ yrs** 1.95 (1.42 - 2.69)  **16-39 yrs** 1.27 (1.01 - 1.61)    ***↑*** *Greater effect in older than younger age group (non-significant)* | Patterson et al (2023) Supplemental Table 8 |
|  | **Maintaining cycling** | Association between living in an intervention area and taking up cycling in 2011 compared with matched control group | **AOR (95% CI)**    **40+ yrs** 1.13 (0.72 – 1.78) p=0.582  **16-39 yrs** 1.24 (0.85 - 1.83) p= 0.268    ***↑*** *Positive effect for older age group (non-significant)* | **AOR (95% CI)**    **40+ yrs** 1.13 (0.72 – 1.78)  **16-39 yrs** 1.24 (0.85 - 1.83)    **↓** *Lesser effect in older than younger age group (non-significant)* | Patterson et al (2023) Supplemental Table 8 |
| **Active travel** | **Cycling or walking as usual commute mode** | DID estimate between 2001 and 2011 intervention towns vs matched comparison towns | **AOR (95% CI)**    **40+ yrs** 1.21 (1.06 - 1.39)  **16-39 yrs** 1.12 (0.99 - 1.28)    **↑** *Positive effect for older age group (significant)* | **AOR (95% CI)**    **40+ yrs** 1.21 (1.06 - 1.39)  **16-39 yrs** 1.12 (0.99 - 1.28)    **↑** *Greater effect in older than younger age group (non-significant)* | Patterson et al (2023) Supplemental Table 7 |
| 3. **ENABLE LONDON** |  |  |  |  |  |
|  | Not available | Not available | Not available | Not available | Not available |
| 4. **iCONNECT** |  |  |  |  |  |
| **Walking** | **Change in walking for transport** | Regression analyses testing intervention effects at two years and interactions to examine whether these effects varied by participant characteristics | **Coefficient (B) from maximally adjusted model (95% CI)**    **65-89 yrs** 4.1 (-9.6 – 17.8) p=0.552  **50-64 yrs** 14.4 (6.1 – 22.66) p=0.001  **35-49 yrs** 3.2 (-10.9 – 17.2) p=0.656  **18-34** **yrs** 15.3 (-7.1 – 37.7) p=0.179    **↑** *Positive effect for all three older age groups (significant in 50-64 yrs)* | **Coefficient (B) from maximally adjusted model (95% CI)**    **65-89 yrs** 4.1 (-9.6 – 17.8)  **50-64 yrs** 14.4 (6.1 – 22.66)  **35-49 yrs** 3.2 (-10.9 – 17.2)  **18-34** **yrs** 15.3 (-7.1 – 37.7  Interaction p=0.934    **↓** *Lesser effect in all three older age groups than youngest age group (non-significant)* | Goodman et al (2014) plus regression data direct from Authors |
|  | **Short-lived increase in walking for transport** | Latent class analysis (LCA) to identify groups whose walking changed in similar ways and multinomial regression to examine factors associated with group membership | Not available | **RRR (95% CI)**    **65-89 yrs** 0.71 (0.24 – 2.06)  **50-64 yrs** 0.82 (0.29 – 2.34)  **30-49 yrs** 0.39 (0.10 – 1.50)  **18-34** **yrs** 1.0 (Ref. category)    **↓** *Lesser effect in all three older age groups than youngest age group (non-significant)* | Panter et al (2017) Table 4 |
|  | **Sustained increase in walking for transport** | As above | Not available | **RRR (95% CI)**    **65-89 yrs** 1.09 (0.36 - 3.33)  **50-64 yrs** 0.91 (0.29 - 2.84)  **30-49 yrs** 1.25 (0.39 - 4.06)  **18-34** **yrs** 1.0 (Ref. category)    **↔** *Mix of lesser effects and greater effects in the three older age groups than youngest age group (non-significant)* | Panter et al (2017) Table 4 |
|  | **Uptake of walking for transport** | As above | Not available | **OR (95% CI)**    **65-89 yrs** 0.39 (0.09 - 1.60)  **50-64 yrs** 1.42 (0.32 - 6.23)  **30-49 yrs** 0.87 (0.18 - 4.18)  **18-34 yrs** 1.0 (Ref. category)  p<0.05 (test for trend)    **↔** *Mix of lesser effects and greater effects in the three older age groups than youngest age group (test for trend significant)* | Panter et al (2017) Table 4 |
| **Cycling** | **Change in cycling for transport** | Regression analyses testing intervention effects at two years and interactions to examine whether these effects varied by participant characteristics | **Coefficient (B) from maximally adjusted model (95% CI)**    **65-89 yrs** 1.6 (-0.7 – 4.0) p=0.162  **50-64 yrs** -1.5 (-4.0 – 0.9) p=0.212  **35-49 yrs** -4.3 (-10.1 – 1.5) p=0.144  **18-34** **yrs** -0.8 (-10.1 – 8.5) p=0.871    **↔** *Mix of positive effects and negative effects for the three older age groups (non-significant)* | **Coefficient (B) from maximally adjusted model (95% CI)**    **65-89 yrs** 1.6 (-0.7 – 4.0)  **50-64 yrs** -1.5 (-4.0 – 0.9)  **35-49 yrs** -4.3 (-10.1 – 1.5)  **18-34** **yrs** -0.8 (-10.1 – 8.5)  Interaction p=0.493    **↔** *Mix of lesser effects and greater effects in the three older age groups than youngest age group (non-significant)* | Goodman et al (2014) plus regression data direct from Authors |
| **Active travel** | **Modal shift from private motor vehicles to walking and cycling after two years in terms of time share** | Logistic regression modelling associations with modal shift in terms of *time* after two years,  with distance to core C2 as measure of exposure | Not available | **Coefficients (t-value)**    **Age** 3.5 x 10^-3^  (1.45)  (Age modelled as a continuous variable, with coefficient representing increase in likelihood per one year increase in age)    **↑** *Greater effect in older than younger age (non-significant)* | Song et al (2017) Table 6 |
|  | **Modal shift from private motor vehicles to walking and cycling after two years in terms of distance share** | Logistic regression modelling associations with modal shift in terms of *distance*,  with distance to core C2 as measure of exposure | Not available | **Coefficients (t-value)**    **Age** -2.5 x 10^-3^  (-0.93)  (Age modelled as a continuous variable, with coefficient representing increase in likelihood per one year increase in age)    **↓** *Lesser effect in older than younger age (non-significant)* | Song et al (2017) Table 6 |
| 5. **MINI HOLLANDS** |  |  |  |  |  |
| **Cycling** | **Past week cycling time** | Regression analyses testing for interactions to examine whether intervention effects after three years varied according to participant characteristics | Not available | Direction of effect data provided to review team by authors    **↑** *Greater effect in older than younger people whether age is categorised as 45+ or younger, or linearly* (non-significant) | Aldred et al, 2019 plus data from Authors |
| **Active travel** | **Past week active travel time** | As above | Not available | Direction of effect data provided to review team by authors    **↑** *Greater effect in older than younger people whether age is categorised as 45+ or younger, or linearly (non-significant)* | Aldred et al (2019) plus data from Authors |

Ethnicity (non-white vs white)

|  | **Outcome** | **Analysis** | **Direction of intervention effect *within* less advantaged population subgroup(s)** | **Direction of effect in less advantaged subgroup(s) *relative to* more advantaged subgroup(s)** | **Data source** |
| --- | --- | --- | --- | --- | --- |
| 1. **CAMBRIDGE GUIDED BUSWAY** |  |  |  |  |  |
|  | Not available | Not available | Not available | Not available | Not available |
| 2. **CYCLING TOWNS** |  |  |  |  |  |
|  | Not available | Not available | Not available | Not available | Not available |
| 3. **ENABLE LONDON** |  |  |  |  |  |
|  | Not available | Not available | Not available | Not available | Not available |
| 4. **iCONNECT** |  |  |  |  |  |
| **Walking** | **Change in walking for transport** | Regression analyses testing intervention effects at two years and interactions to examine whether these effects varied by participant characteristics | **Coefficient (B) from maximally adjusted model (95% CI)**    **Non-white** 24.9 (-68.6 - 118.4) p=0.591  **White** 7.8 (1.8 – 13.9) p=0.011    **↑** *Positive effect for non-white (non-significant)* | **Coefficient (B) from maximally adjusted model (95% CI)**    **Non-white** 24.9 (-68.6 -118.4)  **White** 7.8 (1.8 – 13.9)  Interaction p=0.350    **↑** *Greater effect for in non-white than white (non- significant)* | Goodman et al (2014) plus regression data direct from Authors |
|  | **Short-lived increase in walking for transport** | Latent class analysis (LCA) to identify groups whose walking changed in similar ways and multinomial regression to examine factors associated with group membership | Not available | **RRR (95% CI)**    **Non-white** 1.54 (0.33 - 7.29)  **White** 1.0 (Ref category)    **↑** *Greater effect in non-white than white (non- significant)* | Panter et al (2017) Table 4 |
|  | **Sustained increase in walking for transport** | As above | Not available | **RRR (95% CI)**    **Non-white** 2.21 (0.62 - 7.87)  **White** 1.0 (Ref category)    **↑** *Greater effect in non-white than white (non- significant)* | Panter et al (2017) Table 4 |
|  | **Uptake of walking for transport** | As above | Not available | **OR (95% CI)**    **Non-white** 0.79 (0.27 - 2.26)  **White** 1.0 (Ref category)    **↓** *Lesser effect in non-white than white (non- significant)* | Panter et al (2017) Table 4 |
| **Cycling** | **Change in cycling for transport** | Regression analyses testing intervention effects at two years and interactions to examine whether these effects varied by participant characteristics | **Coefficient (B) from maximally adjusted model (95% CI)**    **Non-white** -13.9 (-36.0 – 8.4) p=0.215  **White** 0.0 (-2.0 - 2.0) p=0.980    **↓** *Negative effect for non-white (non-significant)* | **Coefficient (B) from maximally adjusted model (95% CI)**    **Non-white** -13.9 (-36.0 – 8.4)  **White** 0.0 (-2.0 - 2.0)  Interaction p=0.169    **↓** *Lesser effect in non-white than white (non- significant)* | Goodman et al (2014) plus regression data direct from Authors |
| **Active travel** | **Modal shift from private motor vehicles to walking and cycling after two years in terms of time share** | Logistic regression modelling associations with modal shift in terms of *time* after two years,  with distance to core C2 as measure of exposure | Not available | **Coefficients (t-value)**    **Non-white** -0.69 (-3.12)  **White** (Ref. category)  p=<0.01    **↓** *Lesser effect non-white than white*  *(significant)* | Song et al (2017) Table 6 |
|  | **Modal shift from private motor vehicles to walking and cycling after two years in terms of distance share** | Logistic regression modelling associations with modal shift in terms of *distance*,  with distance to core C2 as measure of exposure | Not available | **Coefficients (t-value)**    **Non-white** -1.18 (-5.15)  **White** (Ref. category)  p=<0.01    **↓** *Lesser effect non-white than white*  *(significant)* | Song et al (2017) Table 6 |
| 5. **MINI HOLLANDS** |  |  |  |  |  |
| **Cycling** | **Past week cycling minutes** | Regression analyses testing for interactions to examine whether intervention effects after three years varied according to participant characteristics | Not available | Direction of effect data provided to review team by authors    **↑** *Greater effect in non-white than white (non-significant)* | Aldred et al (2019) plus data from Authors |
| **Active travel** | **Past week active travel minutes** | As above | Not available | Direction of effect data provided to review team by authors    **↓** *Lesser effect in non-white than white (non-significant)* | Aldred et al (2019) plus data from Authors |

SES: Area based deprivation (high vs low)

|  | **Outcome** | **Analysis** | **Direction of intervention effect *within* less advantaged population subgroup(s)** | **Direction of effect in less advantaged subgroup(s) *relative to* more advantaged subgroup(s)** | **Data source** |
| --- | --- | --- | --- | --- | --- |
| 1. **CAMBRIDGE GUIDED BUSWAY** |  |  |  |  |  |
|  | Not available | Not available | Not available | Not available | Not available |
| 2. **CYCLING TOWNS** |  |  |  |  |  |
| **Walking** | **Walking as usual commute mode** | DID estimate between 2001 and 2011 intervention towns vs matched comparison towns | **AOR (95% CI)**    **High** 1.10 (0.91- 1.33)  **Low** 1.15 (0.97 - 1.37)    **↑** *Positive effect among those living in areas of high deprivation (non-significant)* | **AOR (95% CI)**    **High** 1.10 (0.91 - 1.33)  **Low** 1.15 (0.97 - 1.37)    **↓** *Lesser effect in those living in areas of high deprivation than those living in areas of low deprivation (non-significant)* | Patterson et al (2023) Supplemental Table 7    Similar walking outcome analysis over same time frame in Goodman et al (2013) but less complete numerical data and accompanying interpretation reported so we used Patterson et al (2003) |
|  | **Cycling as usual commute mode** | DID estimate between 2001 and 2011 intervention towns vs matched comparison towns | **AOR (95% CI)**    **High** 1.06 (0.83 - 1.37)  **Low** 0.91 (0.69 - 1.19)    **↑** *Positive effect among those living in areas of high deprivation (non-significant)* | **AOR (95% CI)**    **High** 1.06 (0.83 - 1.37)  **Low** 0.91 (0.69 - 1.19)    **↑** *Greater effect in those living in areas of high deprivation than those living in areas of low deprivation (non-significant)* | Patterson et al (2023) Supplemental Table 7    Similar cycling outcome analysis over same time frame in Goodman et al (2013) but less complete numerical data and accompanying interpretation reported so we used Patterson et al (2003) |
|  | **Taking up cycling** | Association between living in an intervention area and taking up cycling in 2011 compared with matched comparison | **AOR (95% CI)**    **High** 1.42 (1.04 - 1.95) p=0.027  **Low** 1.34 (0.98 - 1.82) p=0.065    **↑** *Positive effect among those living in areas of high deprivation (significant)* | **AOR (95% CI)**    **High** 1.42 (1.04 - 1.95)  **Low** 1.34 (0.98 - 1.82)    **↑** *Greater effect in those living in areas of high deprivation than those living in areas of low deprivation (non-significant)* | Patterson et al (2023) Supplemental Table 8 |
|  | **Maintaining cycling** | Association between living in an intervention area and taking up cycling in 2011 compared with matched comparison | **AOR (95% CI)**    **High** 1.26 (0.78 – 2.01) p=0.345  **Low** 1.17 (0.71 - 1.92) p=0.546    **↑** *Positive effect among those living in areas of high deprivation (non-significant)* | **AOR (95% CI)**    **High** 1.26 (0.78 – 2.01)  **Low** 1.17 (0.71 - 1.92)    **↑** *Greater effect in those living in areas of high deprivation than those living in areas of low deprivation (non-significant)* | Patterson et al (2023) Supplemental Table 8 |
| **Active travel** | **Cycling or walking as usual commute mode** | DID estimate between 2001 and 2011 intervention towns vs matched comparison towns | **AOR (95% CI)**    **High** 1.09 (0.94 - 1.27)  **Low** 1.09 (0.93 - 1.26)    **↑** *Positive effect among those living in areas of high deprivation (non-significant)* | **AOR (95% CI)**    **High** 1.09 (0.94 - 1.27)  **Low** 1.09 (0.93 - 1.26)    **↔** *No difference in effect between those living in areas of high deprivation and those living in areas of low deprivation (non-significant)* | Patterson et al (2023) Supplemental Table 7 |
| 3. **ENABLE LONDON** |  |  |  |  |  |
|  | Not available | Not available | Not available | Not available | Not available |
| 4. **iCONNECT** |  |  |  |  |  |
|  | Not available | Not available | Not available | Not available | Not available |
| 5. **MINI HOLLANDS** |  |  |  |  |  |
|  | Not available | Not available | Not available | Not available | Not available |

SES: Educational level (lower vs highest)

|  | **Outcome** | **Analysis** | **Direction of intervention effect *within* less advantaged population subgroup(s)** | **Direction of effect in less advantaged subgroup(s) *relative to* more advantaged subgroup(s)** | **Data source** |
| --- | --- | --- | --- | --- | --- |
| 1. **CAMBRIDGE GUIDED BUSWAY** |  |  |  |  |  |
| **Active travel** | **Weekly time (in minutes) spent in active commuting**  **(categorised as increase no change, or decrease**) | Regression analyses testing intervention effects at three years post-baseline and interactions to examine whether these effects varied by participant characteristics | **RR (95% CI)**    **Other** Increased 0.89 (0.54 – 1.47) Decreased 1.85 (1.05 – 3.26)  **Degree** Increased 0.85 (0.61 – 1.17) Decreased 0.80 (0.58 – 1.10)    **↓** *Negative effects on AC for ‘other’ (lesser effect on increased AC (non-significant) and greater effect on decreased AC (significant))* | **RR (95% CI)**    **Other** Increased 0.89 (0.54 – 1.47) Decreased 1.85 (1.05 – 3.26)  **Degree** Increased 0.85 (0.61 – 1.17) Decreased 0.80 (0.58 – 1.10)    **↔** *No clear direction of effect;* *greater effect on increased AC in ‘other’ than ‘degree’, but also greater effect on decreased AC in ‘other’ than ‘degree’* | Panter et al (2016) plus regression data direct from Authors |
| 2. **CYCLING TOWNS** |  |  |  |  |  |
| **Walking** | **Walking as usual commute mode** | Difference-in-differences (DID) between 2001 and#2011 intervention towns vs matched comparison towns | **AOR (95% CI)**    **No degree** 1.16 (1.03 - 1.31)  **Degree** 1.19 (0.93 - 1.53)    **↑** *Positive effect for those without a degree (significant)* | **AOR (95% CI)**    **No degree** 1.16 (1.03 - 1.31)  **Degree** 1.19 (0.93 - 1.53)    **↓** *Lesser effect in those without a degree than those with a degree (non-significant)* | Patterson et al (2023) Supplemental Table 7 |
| **Cycling** | **Cycling as usual commute mode** | DID estimate between 2001 and 2011 intervention towns vs matched comparison towns | **AOR (95% CI)**    **No degree** 1.03 (0.84 - 1.25)  **Degree** 1.10 (0.83 - 1.45)    **↑** *Positive effect for those without a degree (non- significant)* | **AOR (95% CI)**    **No degree** 1.03 (0.84 - 1.25)  **Degree** 1.10 (0.83 - 1.45)    **↓** *Lesser effect in those without a degree than those with a degree (non-significant)* | Patterson et al (2023) Supplemental Table 7 |
|  | **Taking up cycling** | Association between living in an intervention area and taking up cycling in 2011 compared with matched control group | **AOR (95% CI)**    **No degree** 1.31 (1.04 - 1.64) p=0.023  **Degree** 1.84 (1.31 - 2.59) p=<0.001    **↑** *Positive effect for those without a degree (significant)* | **AOR (95% CI)**    **No degree** 1.31 (1.04 - 1.64)  **Degree** 1.84 (1.31 - 2.59)    **↓** *Lesser effect in those without a degree than those with a degree (non-significant)* | Patterson et al (2023) Supplemental Table 8 |
|  | **Maintaining cycling** | Association between living in an intervention area and taking up cycling in 2011 compared with matched control group | **AOR (95% CI)**    **No degree** 1.07 (0.76 - 1.51) p=0.713  **Degree** 1.81 (1.01 – 3.26) p=0.046    **↑** *Positive effect for those without a  degree (non-significant)* | **AOR (95% CI)**    **No degree** 1.07 (0.76 - 1.51)  **Degree** 1.81 (1.01 – 3.26)    **↓** *Lesser effect in those without a degree than those with a degree (non-significant)* | Patterson et al  (2023) Supplemental Table 8 |
| **Active travel** | **Cycling or walking as usual commute mode** | DID estimate between 2001 and 2011 intervention towns vs matched comparison towns | **AOR (95% CI)**    **No degree** 1.13 (1.02 - 1.26)  **Degree** 1.18 (0.97 - 1.44)    **↑** *Positive effect for those without a degree (significant)* | **AOR (95% CI)**    **No degree** 1.13 (1.02 - 1.26)  **Degree** 1.18 (0.97 - 1.44)    **↓** *Lesser effect in those without a degree than those with a degree (non-significant)* | Patterson et al (2023) Supplemental Table 7 |
| 3. **ENABLE LONDON** |  |  |  |  |  |
|  | Not available | Not available | Not available | Not available | Not available |
| 4. **iCONNECT** |  |  |  |  |  |
| **Walking** | **Change in walking for transport** | Regression analyses testing intervention effects at two years and interactions to examine whether these effects varied by participant characteristics | **Coefficient (B) from maximally adjusted model (95% CI)**    **None/other** 4.9 ( -10.1 – 19.9) p=0.521  **Secondary** 11.3 (-0.7 – 23.3) p=0.065  **Tertiary** 9.4 (1.5 – 17.3) p=0.020    **↑** *Positive effect for both lower education level groups (non-significant)* | **Coefficient (B) from maximally adjusted model (95% CI)**    **None/other** 4.9 (-10.1 – 19.9)  **Secondary** 11.3 (-0.7 – 23.3)  **Tertiary** 9.4 (1.5 – 17.3)  Interaction p=0.933    **↔** *Mix of lesser and greater effects in the two lower education level groups than in the highest education level group (non-significant)* | Goodman et al (2014) plus regression data direct from Authors |
|  | **Short-lived increase in walking for transport** | Latent class analysis (LCA) to identify groups whose walking changed in similar ways and multinomial regression to examine factors associated with group membership | Not available | **RRR (95% CI)**    **Lower than secondary**  1.36 (0.54 - 3.44)  **Secondary or higher**  1.61 (0.72 - 3.58)  **Tertiary** 1.0 (Ref category)  p=<0.05 (test for trend)    **↑** *Greater effect in the two lower education level groups than in the highest education level group (test for trend significant)* | Panter et al (2017) Table 4 |
|  | **Sustained increase in walking for transport** | As above | Not available | **RRR (95% CI)**    **Lower than secondary**  3.21 (1.48 - 6.94)  **Secondary or higher**  1.15 (0.52 - 2.58)  **Tertiary** 1.0 (Ref category)  p=<0.01 (test for trend)    **↑** *Greater effect in the two lower education level groups than in the highest education level group (test for trend significant)* | Panter et al (2017) Table 4 |
|  | **Uptake of walking for transport** | As above | Not available | **OR (95% CI)**    **Lower than secondary**  0.76 (0.45 - 1.29)  **Secondary or higher**  0.50 (0.31 - 0.82)  **Tertiary** 1.0 (Ref category)  p=<0.05 (test for trend)    **↓** *Lesser effect in the two lower education level groups than in the highest education level group (test for trend significant)* | Panter et al (2017) Table 4 |
| **Cycling** | **Change in cycling for transport** | Regression analyses testing intervention effects at two years and interactions to examine whether these effects varied by participant characteristics | **Coefficient (B) from maximally adjusted model (95% CI)**    **None/other** -2.1 (-5.0 – 0.7) p=0.139  **Secondary** -0.9 (-3.3 – 1.5) p=0.461  **Tertiary** 1.4 (-2.9 – 5.6) p=0.524    **↓** *Negative effect for both lower education level groups (non-significant)* | **Coefficient (B) from maximally adjusted model (95% CI)**    **None/other** -2.1 (-5.0 – 0.7)  **Secondary** -0.9 (-3.3 – 1.5)  **Tertiary** 1.4 (-2.9 – 5.6)  Interaction p=0.324    **↓** *Lesser effects in the two lower education level groups than in the highest education level group (non-significant)* | Goodman et al (2014) plus regression data direct from Authors |
| **Active travel** | **Modal shift from private motor vehicles to walking and cycling after two years in terms of time share** | Logistic regression modelling associations with modal shift in terms of *time* after two years,  with distance to core C2 as measure of exposure | Not available | **Coefficients (t-value)**    **Less than university degree** (Ref. category)  **Degree or higher** 0.81 (9.69)  p=<0.01    **↓** *Lesser effect in those with less than university degree education than those with (significant)* | Song et al (2017) Table 6 |
|  | **Modal shift from private motor vehicles to walking and cycling after two years in terms of distance share** | Logistic regression modelling associations with modal shift in terms of *distance*,  with distance to core C2 as measure of exposure | Not available | **Coefficients (t-value)**    **Less than university degree** (Ref. category)  **Degree or higher** 0.48 (5.19)  p=<0.01    **↓** *Lesser effect in those with less than university degree education than those with (significant)* | Song et al (2017) Table 6 |
| 5. **MINI HOLLANDS** |  |  |  |  |  |
|  | Not available | Not available | Not available | Not available | Not available |

SES: Housing status/tenure (social/other vs owned/market rent)

|  | **Outcome** | **Analysis** | **Direction of intervention effect *within* less advantaged population subgroup(s)** | **Direction of effect in less advantaged subgroup(s) *relative to* more advantaged subgroup(s)** | **Data source** |
| --- | --- | --- | --- | --- | --- |
| 1. **CAMBRIDGE GUIDED BUSWAY** |  |  |  |  |  |
| **Active travel** | **Weekly time (in minutes) spent in active commuting**  **(categorised as increase no change, or decrease**) | Regression analyses testing intervention effects at three years post-baseline and interactions to examine whether these effects varied by participant characteristics | **RR (95% CI)**    **Rented/other** Increased 3.32 (0.82 – 13.37) Decreased 2.64 (0.91 – 7.69)  **Own home** Increased 1.30 (0.96 – 1.76) Decreased 0.94 (0.63 – 1.41)    **↔** *Mix of positive effects (greater effect on increased active commuting (AC)) and negative effects (greater effect on decreased AC) for ‘rented/other’ (both non-significant)* | **RR (95% CI)**    **Rented/other** Increased 3.32 (0.82 – 13.37) Decreased 2.64 (0.91 – 7.69)  **Own home** Increased 1.30 (0.96 – 1.76) Decreased 0.94 (0.63 – 1.41)    **↔** *No clear direction of effect;* *(greater effect on increased AC for ‘rented/other’ than ‘own home’ but also greater effect on decreased AC in ‘rented/other than ‘own home’* | Panter et al (2016) plus regression data direct from Authors |
| 2. **CYCLING TOWNS** |  |  |  |  |  |
|  | Not available | Not available | Not available | Not available | Not available |
| 3. **ENABLE LONDON** |  |  |  |  |  |
| **Walking** | **Change in daily walking (in minutes) in intervention group relative to change in control group** | Change in average daily minutes from baseline to follow-up examined using multilevel linear regression models | **Difference (95% CI)**    **Social housing** -1.8 (-8.4 - 4.8) p=0.59  **Intermediate housing** −1.9 (−7.5 - 3.8) p=0.52  **Market-rent** -4.2 (−15.4 - 7.0) p= 0.46    **↓** *Negative effect for both social and intermediate housing (non-significant)* | **Difference (95% CI)**    **Social housing** -1.8 (-8.4 - 4.8)  **Intermediate housing** −1.9 (−7.5 - 3.8)  **Market-rent** -4.2 (−15.4 - 7.0)    **↑** *Greater effect in both social and intermediate housing than in market-rent housing (non-significant)* | Limb et al  (2020) Table 3 |
| **Cycling** | **Change in daily cycling (in minutes) in intervention group relative to change in control group** | Change in average daily minutes from baseline to follow-up examined using multilevel linear regression models | **Difference (95% CI)**    **Social housing** 0.3 (-0.8 - 1.4) p=0.58  **Intermediate housing** 1.0 (−1.6 - 3.7) p=0.44  **Market-rent** 1.9 (-3.6 - 7.4) p=0.50    **↑** *Positive effect for both social and intermediate housing (non-significant)* | **Difference (95% CI)**    **Social housing** 0.3 (-0.8 - 1.4)  **Intermediate housing** 1.0 (−1.6 - 3.7)  **Market-rent** 1.9 (-3.6 - 7.4)    **↓** *Lesser effect in both social and intermediate housing than in market-rent housing (non-significant)* | Limb et al  (2020) Table 3 |
| **Active travel** | **Change in daily walking/cycling (in minutes) in intervention group relative to change in control group** | Change in average daily minutes from baseline to follow-up examined using multilevel linear regression models | **Difference (95% CI)**    **Social housing** -1.5 (-8.4 - 5.4) p=0.68  **Intermediate housing** −0.9 (−7.1 - 5.4) p=0.78  **Market-rent** -2.4 (-16.0 - 11.3) p=0.74    **↓** *Negative effect for both social and intermediate housing (non-significant)* | **Difference (95% CI)**    **Social housing** -1.5 (-8.4 - 5.4)  **Intermediate housing** −0.9 (−7.1 - 5.4)  **Market-rent** -2.4 (-16.0 - 11.3)    **↑** *Greater effect in both social and intermediate housing than in market-rent housing (non-significant)* | Limb et al  (2020) Table 3 |
| **Health** | **Change BMI in intervention group relative to change in control group** | Change in BMI from baseline to follow-up examined using multilevel linear regression models | **Difference (95% CI)**    **Social housing** 0.2 (–0.4 - 0.8) p=0.49  **Intermediate housing** 0.1 (–0.3 - 0.5) p=0.66  **Market-rent** 0.2 (–0.4 - 0.8) p=0.52    **↓** *Negative effect (i.e. increased BMI) for both social and intermediate housing (non-significant)* | **Difference (95% CI)**    **Social housing** 0.2 (–0.4 - 0.8)  **Intermediate housing** 0.1 (–0.3 - 0.5)  **Market-rent** 0.2 (–0.4 - 0.8)    **↔** *Mix of same and lesser effect on BMI in both social and intermediate housing than in market-rent housing (non-significant)* | Owen et al (2020) Table 8 |
|  | **Change % body fat in intervention group relative to change in control group** | Change in % body fat from baseline to follow-up examined using multilevel linear regression models | **Difference (95% CI)**    **Social** –0.3 (–1.3 - 0.7) p=0.60  **Intermediate housing** 0.2 (–0.6 - 0.9) p=0.65  **Market-rent** 0.4 (–1.0 - 1.8) p=0.58    **↔** *Mix of positive effect for social housing (i.e. lower % body fat) and negative effect for intermediate housing (i.e. higher % body fat) (non-significant)* | **Difference (95% CI)**    **Social** –0.3 (–1.3 - 0.7)  **Intermediate housing** 0.2 (–0.6 - 0.9)  **Market-rent** 0.4 (–1.0 - 1.8)    **↑** *Greater effect on % body fat (i.e. lower % body fat) in both social and intermediate housing than in market-rent housing (non-significant)* | Owen et al (2020) Table 8 |
| 4. **iCONNECT** |  |  |  |  |  |
|  | Not available | Not available | Not available | Not available | Not available |
| 5. **MINI HOLLANDS** |  |  |  |  |  |
|  | Not available | Not available | Not available | Not available | Not available |

SES: Household income (lower vs highest)

|  | **Outcome** | **Analysis** | **Direction of intervention effect *within* less advantaged population subgroup(s)** | **Direction of effect in less advantaged subgroup(s) *relative to* more advantaged subgroup(s)** | **Data source** |
| --- | --- | --- | --- | --- | --- |
| 1. **CAMBRIDGE GUIDED BUSWAY** |  |  |  |  |  |
|  | Not available | Not available | Not available | Not available | Not available |
| 2. **CYCLING TOWNS** |  |  |  |  |  |
|  | Not available | Not available | Not available | Not available | Not available |
| 3. **ENABLE LONDON** |  |  |  |  |  |
|  | Not available | Not available | Not available | Not available | Not available |
| 4. **iCONNECT** |  |  |  |  |  |
| **Walking** | **Change in walking for transport** | Regression analyses testing intervention effects at two years and interactions to examine whether these effects varied by participant characteristics | **Coefficient (B) from maximally adjusted model (95% CI)**    **≤£20,000** 14.2 (-1.9 – 30.2) p=0.082  **£20,001-£40,000** 3.7 (-5.4 – 12.7) p=0.428  **>£40,000** 8.0 (-3.3 – 19.2) p=0.162    **↑** *Positive effect for the two lower income groups (non-significant)* | **Coefficient (B) from maximally adjusted model (95% CI)**    **≤£20,000** 14.2 (-1.9 – 30.2)  **£20,001-£40,000** 3.7 (-5.4 – 12.7)  **>£40,000** 8.0 (-3.3 – 19.2)  Interaction p=0.548    **↔** *Mix of lesser and greater effects in the two lower income groups than in the highest income group (non-significant)* | Goodman et al (2014) plus regression data direct from Authors |
|  | **Short-lived increase in walking for transport** | Latent class analysis (LCA) to identify groups whose walking changed in similar ways and multinomial regression to examine factors associated with group membership | Not available | **RRR (95% CI)**    **≤£20,000**  3.43 (1.26 – 9.33)  **£20,001-£40,000**  1.92 (0.69 – 5.33)  **>£40,000** 1.0 (Ref category)  p=<0.01 (test for trend)    **↑** *Greater effect in the two lower income groups than in the highest income group (test for trend significant)* | Panter et al (2017) Table 4 |
|  | **Sustained increase in walking for transport** | As above | Not available | **RRR (95% CI)**    **≤£20,000**  3.79 (1.55 – 9.26)  **£20,001-£40,000)**  2.41 (1.00 – 5.82)  **>£40,000** 1.0 (Ref category)  p=<0.01 (test for trend)    **↑** *Greater effect in the two lower income groups than in the highest income group (test for trend significant)* | Panter et al (2017) Table 4 |
|  | **Uptake of walking for transport** | As above | Not available | **OR (95% CI)**    **≤£20,000**  0.74 (0.42 – 1.31)  **£20,001-£40,000**  1.17 (0.71 – 1.93)  **>£40,000** 1.0 (Ref category)    **↔** *Mix of lesser and greater effects in the two lower income groups than in the highest income group (non-significant)* | Panter et al (2017)Table 4 |
| **Cycling** | **Change in cycling for transport** | Regression analyses testing intervention effects at two years and interactions to examine whether these effects varied by participant characteristics | Coefficient (B) from maximally adjusted model (95% CI)    **≤£20,000** -0.8 (-3.5 – 1.9) p=0.548  **£20,001-£40,000** 0.4 (-2.3 – 3.1) p=0.767  **>£40,000** -1.0 (-4.7 – 2.7) p=0.586    **↔** *Mix of positive and negative effects for the two lower income groups (non-significant)* | Coefficient (B) from maximally adjusted model (95% CI)    **≤£20,000** -0.8 (-3.5 – 1.9)  **£20,001-£40,000** 0.4 (-2.3 – 3.1)  **>£40,000** -1.0 (-4.7 – 2.7)  Interaction p=0.591    **↑** *Greater effects in the two lower income groups than in the highest income group (non-significant)* | Goodman et al (2014) plus regression data direct from Authors |
| 5. **MINI HOLLANDS** |  |  |  |  |  |
|  | Not available | Not available | Not available | Not available | Not available |

SES: Employment status (not working full time/retired vs working/student)

|  | **Outcome** | **Analysis** | **Direction of intervention effect *within* less advantaged population subgroup(s)** | **Direction of effect in less advantaged subgroup(s) *relative to* more advantaged subgroup(s)** | **Data source** |
| --- | --- | --- | --- | --- | --- |
| 1. **CAMBRIDGE GUIDED BUSWAY** |  |  |  |  |  |
|  | Not available | Not available | Not available | Not available | Not available |
| 2. **CYCLING TOWNS** |  |  |  |  |  |
|  | Not available | Not available | Not available | Not available | Not available |
| 3. **ENABLE LONDON** |  |  |  |  |  |
|  | Not available | Not available | Not available | Not available | Not available |
| 4. **iCONNECT** |  |  |  |  |  |
| **Walking** | **Change in walking for transport** | Regression analyses testing intervention effects at two years and interactions to examine whether these effects varied by participant characteristics | **Coefficient (B) from maximally adjusted model (95% CI)**    **Other** 17.6 (-10.3 – 45.6) p=0.213  **Retired** 5.4 (-5.6 – 16.5) p=0.333  **Student** -105.7 (-493.5 – 282.1) p=0.572  **Working** 10.9 (2.5 – 19.3) p=0.011    **↑** *Positive effect for both ‘other’ and ‘retired’ groups (non-significant)* | **Coefficient (B) from maximally adjusted model (95% CI)**    **Other** 17.6 (-10.3 – 45.6)  **Retired** 5.4 (-5.6 – 16.5)  **Student** -105.7 (-493.5 – 282.1)  **Working** 10.9 (2.5 – 19.3)  Interaction p=0.868    **↔** *Mix of lesser and greater effects in the ‘other’ and ‘retired’ groups than in the ‘working’ and ‘student’ groups (non-significant)* | Goodman et al (2014) plus regression data direct from Authors |
|  | **Short-lived increase in walking for transport** | Latent class analysis (LCA) to identify groups whose walking changed in similar ways and multinomial regression to examine factors associated with group membership | Not available | **RRR (95% CI)**    **Unemployed/other/sick** 0.84 (0.19 - 3.74)  **Retired** 2.59 (0.96 – 6.95)  **Working/student** 1.0 (Ref category)    **↔** *Mix of lesser and greater effects in the ‘unemployed/other/sick’ and ‘retired’ groups than in the ‘working/student’ group (non-significant)* | Panter et al (2017) Table 4 |
|  | **Sustained increase in walking for transport** | As above | Not available | **RRR (95% CI)**    **Unemployed/other/sick** 1.94 (0.70 – 5.38)  **Retired** 2.85 (1.10 – 7.40)  **Working/student** 1.0 (Ref category)    **↑** *Greater effects in the ‘unemployed/other/sick’ and ‘retired’ groups than in the ‘working/student’ group (non-significant)* | Panter et al (2017) Table 4 |
|  | **Uptake of walking for transport** | As above | Not available | **OR  (95% CI)**    **Unemployed/other/sick** 1.15 (0.46 – 2.85)  **Retired** 1.10 (0.60 – 2.00)  **Working/student** 1.0 (Ref category)    **↑** *Greater effects in the ‘unemployed/other/sick’ and ‘retired’ groups than in the ‘working/student’ group (non-significant)* | Panter et al (2017) Table 4 |
| **Cycling** | **Change in cycling for transport** | Regression analyses testing intervention effects at two years and interactions to examine whether these effects varied by participant characteristics | **Coefficient (B) from maximally adjusted model (95% CI)**    **Other** -9.2 (-18.8 – 0.5) p=0.062  **Retired** 1.3 (-0.9 – 3.5) p=0.252  **Student** -10.5 (-83.2 – 62.2) p=0.765  **Working** -0.9 (-4.0 – 2.3) p=0.576    **↔** *Mix of negative and positive effects for both ‘other’ and ‘retired’ groups (non-significant)* | **Coefficient (B) from maximally adjusted model (95% CI)**    **Other** -9.2 (-18.8 – 0.5)  **Retired** 1.3 (-0.9 – 3.5)  **Student** -10.5 (-83.2 – 62.2)  **Working** -0.9 (-4.0 – 2.3)  Interaction p=0.636    **↔** *Mix of lesser and greater effects in the ‘other’ and ‘retired’ groups than in the ‘working’ and ‘student’ groups (non-significant)* | Goodman et al (2014) plus regression data direct from Authors |
| 5. **MINI HOLLANDS** |  |  |  |  |  |
| **Cycling** | **Past week cycling minutes** | Regression analyses testing for interactions to examine whether intervention effects after three years varied according to participant characteristics | Not available | Direction of effect data provided to review team by authors    **↑** *Greater effect in ‘not full time employed’ than in ‘full-time employed’ (non-significant)* | Aldred et al  (2019) plus data from Authors |
| **Active travel** | **Past week active travel minutes** | As above | Not available | Direction of effect data provided to review team by authors    **↑** *Greater effect in ‘not full time employed’ than in ‘full-time employed’ (non-significant)* | Aldred et al (2019) plus data from Authors |

SES: Social grade (lower vs highest)

|  | **Outcome** | **Analysis** | **Direction of intervention effect *within* less advantaged population subgroup(s)** | **Direction of effect in less advantaged subgroup(s) *relative to* more advantaged subgroup(s)** | **Data source** |
| --- | --- | --- | --- | --- | --- |
| 1. **CAMBRIDGE GUIDED BUSWAY** |  |  |  |  |  |
|  | Not available | Not available | Not available | Not available | Not available |
| 2. **CYCLING TOWNS** |  |  |  |  |  |
| **Cycling** | Any cycling in a typical week in the previous year | Proportion of survey participants, 2009 compared to 2006 (baseline) | **Percentages**    **E** *% higher in 2009 than 2006*  **D** *% higher in 2009 than 2006*  **C** *% higher in 2009 than 2006*  **B** *% higher in 2009 than 2006*  **A** *% higher in 2009 than 2006*    **↑** *Positive effect for all four lower social grade groups (E, D, C, and B) (significance data not available)* | **Change in percentages**    **E** ~Same as in group A  **D** Lesser than in group A  **C** ~Same as in group A  **B** Greater than in group A  **A** (Ref.  group)    **↔** *Mix of lesser and greater effects in the four lower social grade groups (E, D, C, and B) than in the highest social grade group (A) (significance data not available)* | Sloman et al (2009)  Data extracted from Figure 4, page 13 (exact numeric data not provided) |
| 3. **ENABLE LONDON** |  |  |  |  |  |
|  | Not available | Not available | Not available | Not available | Not available |
| 4. **iCONNECT** |  |  |  |  |  |
|  | Not available | Not available | Not available | Not available |  |
| 6. **MINI HOLLANDS** |  |  |  |  |  |
|  | Not available | Not available | Not available | Not available | Not available |

**S5: Use of London-York PRP Evidence Review Facility equity checklist**

During development of the review protocol, we applied the current version (v1.0) of this checklist (Shemilt et al, 2024; Available from: <https://eppi.ioe.ac.uk/cms/Default.aspx?tabid=3939>). This checklist (which as of March 2026 remains in ongoing development) has been developed within the London-York PRP Evidence Review Facility as a tool intended to support routine and systematic consideration and reporting of methods and processes for incorporating health equity perspectives and/or investigating health equity issues in evidence syntheses. It incorporates a series of signalling questions and linked guidance depending on responses. The key implications of completing this checklist for this specific review – with our responses included in full in the Box immediately below – were that we explicitly aimed to identify, synthesise and report, in accordance with PRISMA-Equity (Welch et al, 2012), the evidence in relation to each of our specified population subgroup foci, i.e. the evidence concerning each of sex/gender; ethnicity; age; and socioeconomic status.

**Provisional assessment of equity considerations at protocol stage**

The current checklist (v1.0; Shemilt et al, 2024) comprises a set of six signalling questions, and we considered in turn whether these potentially apply to studies eligible for inclusion in this review:

**1a. Is the intervention under investigation targeted at specific marginalised, at-risk, socially excluded and/or inclusion health group(s) of people?**

*We judged this as sufficiently known and/or plausible to justify a ‘Yes’ response across all population groups that were within the focus of the review i.e. those relating to sex/gender; ethnicity; age; and socioeconomic status. This judgement is informed by prior scoping and evidence mapping work for DHSC identifying a substantive body of evidence evaluating active travel interventions targeted at specific disadvantaged population subgroups.*

**1b. Is the intervention under investigation aimed at reducing social gradients across populations or among subgroups of the population?**

*We judged this as sufficiently known and/or plausible to justify a ‘Yes’ response across all groups.*

*The implication of our responses to questions 1a and 1b, after following the corresponding decision tree, is that PRISMA-E should be used (in conjunction with PRISMA and PRISMA-P) for this review, as should PRO-EDI (if available, as it is currently being developed).*

**2a. Is the health condition, public health issue, or phenomenon, being addressed by the review (and/or map) more likely to be experienced by one or more specific marginalised, socially excluded and/or inclusion health group(s) of people?**

*Given there appears to be some evidence for each of the set of population subgroups specified for this review that membership may be associated with reduced active travel (Sustrans et al, 2022), we considered this to be ‘Yes’ in relation to groups concerning: sex/gender, ethnicity, age, and socioeconomic status (e.g. categorisation via education, income, occupational status, geography), each of which we specify we intend to examine in this review.*

**2b. Are aspects of the intervention(s) and/or comparator(s), including how they are provided, expected to make it harder for some specific marginalised, at-risk, socially excluded and/or inclusion health group(s) of people to take part in eligible studies?**

*We judged ‘Unsure’ to be the most appropriate response across all groups. We do know at outset that at least some of the eligible studies specifically target these groups to take part, meaning that at least in these studies these groups are not clearly disadvantaged from taking part in eligible studies. However, this does not address relative difficulty of participation, and at outset we are not aware of specific evidence on the participation of these groups relative to that of less disadvantaged groups.*

**2c. Are elements of study design, such as eligibility criteria or recruitment and consent processes, expected to make it harder for some specific marginalised, at-risk, socially excluded and/or inclusion health group(s) of people to take part in eligible studies?**

*We judged ‘Unsure’ to be the most appropriate response across all groups. We do know at outset that at least some of the eligible studies specifically target these groups to take part, meaning that at least in these studies these groups are not clearly disadvantaged from taking part in eligible studies. However, this does not address relative difficulty of participation, and at outset we are not aware of specific evidence on the participation of these groups relative to that of less disadvantaged groups.*

*The implications of our responses to questions 2a, 2b and 2c, after following the corresponding decision tree, are as follows:*

*PRISMA-E should be used (in conjunction with PRISMA and PRISMA-P) for this review, as should PRO-EDI (if available, as it is currently being developed); a ‘Characteristics of Included Studies’ table should be used; where possible and appropriate, data on the numbers and percentages of participants from each specified subgroup in a) each study sample and b) in total across all studies included in the review should be extracted and reported; such data assessing the extent of applicability of review findings to target populations (e.g. highlighting over- and under-representation) should be summarised and discussed, including being used when interpreting data as well as highlighting related uncertainty; related data should be considered when assessing ‘indirectness’ in the planned GRADE assessment; should any logic models be developed for the review, theory/evidence regarding the potential impacts of inequities in access, participation, or involvement in interventions should be considered for inclusion in that model*

**3. Are the impacts of, or responses to, the intervention(s) / exposure(s), or the experiences of the phenomenon, under investigation, expected to differ among specific marginalised, at-risk, socially excluded and/or inclusion health group(s) of people in important or meaningful ways?**

*This signalling question necessitated a judgement on how confident we are that effects differ, which we considered to be ‘Unsure (whether effects differ)’ for all groups. This is because known evidence from multiple reviews of the patterning of intervention impacts for different population subgroups (e.g. Hansmann et al, 2022; Medeiros et al, 2021; Tcymbal et al, 2020, Smith et al, 2017), suggested no clearly consistent and reliable differences in impacts. The principal justification for conducting the current review is that we are not currently confident about whether effects differ.*

*The implication of our response to question 3, after following the corresponding decision tree, is that the review will be reported in accordance with PRISMA-E. In line with this, we intend at outset to conduct and report separate sets of analyses by specified population subgroups and for each of those we intend to consider absolute and relative effects in our analyses (where possible). However, we were unable to identify and justify further more specific actions in line with the decision tree for this signalling question due to the degree of uncertainty both about whether effects differ (which as mentioned is the main justification for the review) and the considerable uncertainty about the methods we will ultimately apply given we know at outset that the included data will be particularly challenging to synthesise.*
